# Target Inhibition Networks: Predicting Selective Combinations of Druggable Targets to Block Cancer Survival Pathways

**DOI:** 10.1371/journal.pcbi.1003226

**Published:** 2013-09-12

**Authors:** Jing Tang, Leena Karhinen, Tao Xu, Agnieszka Szwajda, Bhagwan Yadav, Krister Wennerberg, Tero Aittokallio

**Affiliations:** Institute for Molecular Medicine Finland (FIMM), University of Helsinki, Helsinki, Finland; University of Toronto and Mount Sinai Hospital, Canada

## Abstract

A recent trend in drug development is to identify drug combinations or multi-target agents that effectively modify multiple nodes of disease-associated networks. Such polypharmacological effects may reduce the risk of emerging drug resistance by means of attacking the disease networks through synergistic and synthetic lethal interactions. However, due to the exponentially increasing number of potential drug and target combinations, systematic approaches are needed for prioritizing the most potent multi-target alternatives on a global network level. We took a functional systems pharmacology approach toward the identification of selective target combinations for specific cancer cells by combining large-scale screening data on drug treatment efficacies and drug-target binding affinities. Our model-based prediction approach, named TIMMA, takes advantage of the polypharmacological effects of drugs and infers combinatorial drug efficacies through system-level target inhibition networks. Case studies in MCF-7 and MDA-MB-231 breast cancer and BxPC-3 pancreatic cancer cells demonstrated how the target inhibition modeling allows systematic exploration of functional interactions between drugs and their targets to maximally inhibit multiple survival pathways in a given cancer type. The TIMMA prediction results were experimentally validated by means of systematic siRNA-mediated silencing of the selected targets and their pairwise combinations, showing increased ability to identify not only such druggable kinase targets that are essential for cancer survival either individually or in combination, but also synergistic interactions indicative of non-additive drug efficacies. These system-level analyses were enabled by a novel model construction method utilizing maximization and minimization rules, as well as a model selection algorithm based on sequential forward floating search. Compared with an existing computational solution, TIMMA showed both enhanced prediction accuracies in cross validation as well as significant reduction in computation times. Such cost-effective computational-experimental design strategies have the potential to greatly speed-up the drug testing efforts by prioritizing those interventions and interactions warranting further study in individual cancer cases.

## Author Summary

Selective inhibition of specific panels of multiple protein targets provides an unprecedented potential for improving therapeutic efficacy of anticancer agents. We introduce a computational systems pharmacology strategy, which uses the concept of target inhibition networks to predict effective multi-target combinations for treating specific cancer types. The strategy is based on integration of two complementary information sources, drug treatment efficacies and drug-target binding affinities, which are readily available in drug screening labs. Compared to the cancer sequencing efforts, which often result in a huge number of non-targetable genetic alterations, the target combinations from our strategy are druggable, by definition, hence enabling more straightforward translation toward clinically actionable treatment strategies. The model predictions were experimentally validated using siRNA-mediated target silencing screens in three case studies involving MDA-MB-231 and MCF-7 breast cancer and BxPC-3 pancreatic cancer cells. In more general terms, the cancer cell-specific target inhibition networks provided additional insights into the drugs' mechanisms of action, for instance, how the cancer cell survival pathways can be targeted by synergistic and synthetic lethal interactions through multi–target perturbations. These results demonstrate that the principles introduced here offer the possibilities to move toward more systematic prediction and evaluation of the most effective drug target combinations.

## Introduction

Over the past decade, there has been a significant increase in the R&D cost when translating new cancer drug candidates into effective therapies in the clinic. The two single most important reasons are (*i*) lack of efficacy and (*ii*) clinical safety of the candidate drug compounds [1]. This decline in productivity of the pharmaceutical industry has greatly challenged the dominant paradigm in drug discovery, where such ‘magic bullet’ compounds are being searched that could produce dramatic treatment outcomes at a population-level by modulating one specific target only. The shortcomings of such ‘one-size-fits-all’ treatment strategies are well reflected in the disappointing outcome of the current anticancer drug development, where agents directed at an individual target often show limited efficacy and safety due to factors such as off-target activities, network robustness, bypass mechanisms and cross-talk across compensatory escape pathways [2]–[4]. Most cancers develop from specific combinations of genetic alterations accumulated in tumor cells, which are often distinct between different cancer types and result in different treatment responses to the same therapy. Moreover, the extensive mutational heterogeneity results in alterations within multiple molecular pathways, making most advanced tumors readily resistant to single-targeted agents. Therefore, rational strategies to develop multi-targeted therapies for specific cancer types are needed to attack the resistance problem and to provide more effective and personalized treatment strategies [5]. Targeted drug combinations may also overcome the side effects associated with high doses of single drugs by countering pathway compensation and thereby increasing cancer cell killing while minimizing overlapping toxicity and allowing reduced dosage of each compound [6].

Even though it is widely acknowledged that effective cancer treatments need to go beyond the traditional ‘one disease, one drug, one target’ paradigm, the major bottleneck hindering the development of combinatorial therapies is the lack of such systematic experimental-computational approaches that could pinpoint the most effective combinations [7]–[9]. While efforts based on next-generation sequencing are very successful at systematically characterizing the structural basis of cancers, by identifying the genomic mutations associated with each cancer type [10], these findings often do not lead to clinically actionable therapeutic strategies and rarely to rational targeted combinations. The large number of genetic alterations present in tumor cells makes the discrimination of the cancer-specific driver mutations and pathways highly challenging, and even when genetic aberrations with pathogenetic importance can be identified, these may not be pharmaceutically actionable. Moreover, genes not altered at the genomic level may also play essential roles in the cancer progression, hence providing additional therapeutic opportunities [11]. In contrast, systematic assessment of genes for their contribution to tumor addictions can provide functional insight into the molecular mechanisms and pathways behind specific cancer types, hence highlighting their vulnerabilities associated with driver genes, synthetic lethal interactions and other tumor dependencies [12]–[14], which are complementary to the structural information obtained from the cancer mutational landscape. Advances in high-throughput chemical and RNAi screening have now made it possible to carry out comprehensive functional screening in cancer cells, providing novel targets for the next generation of anticancer therapies for patients sharing a common genetic background [15]–[18].

However, despite the emerging possibilities for perturbing gene functions with a wide spectrum of shRNA/siRNA libraries or using diverse drug and compound collections, functional interactions between genes and/or drugs have remained extremely difficult to predict on a global scale [18]. The complex genotype-phenotype relationships behind such interactions pose modeling challenges beyond the reach of the classical linear approaches. Moreover, polypharmacologic compounds elicit their bioactivities by modulating multiple targets, which leads to a combinatorial explosion both in the pharmacological and molecular spaces. Taken together, the exponentially increasing number of possible RNAi, chemical, target and dose combinations poses great experimental challenges, and exhaustive experimentation with all the possible combinations is impossible in practice, making the pure experimental approach quickly unfeasible [19]. To meet these computational and experimental challenges, novel modeling frameworks and efficient computational algorithms are needed to effectively reduce the search space for determining the most promising combinations and prioritizing their experimental evaluation. Ideally, the experimental setup should be both economical and practical, utilizing such functional measurements and phenotypic readouts that are readily available in typical drug screening experiments. Moreover, the experimental and computational platforms should also be compatible with the eventual clinical translation in the sense that the measurements and their analysis can be made in each patient individually, and that the modeling and algorithmic predictions can be calculated in a reasonable time.

A number of computational algorithms have been developed for predicting drug combinations *in silico* [5], [9], [20]. Most of the approaches are based on detailed mathematical modeling, utilizing *a priori* knowledge extracted from databases, such as those focusing on established cancer pathways, metabolic network constructions or literature-curated models [21]–[23]. A limitation of such detailed models is that global kinetic information for many cancer-related systems are still rarely available, and reduced subsystem models are often biased toward what is already known about the cancer processes. For instance, pathway-specific models may miss important novel features, such as pathway cross-talks or novel cancer dependencies. Accordingly, although major canonical pathways involved in different cancer types are increasingly well established, individual pathway models cannot capture the complex and context-dependent cellular wiring patterns behind distinct cancer phenotypes [5]. There are also approaches that take the cell context into account by means of global gene expression or targeted phosphoproteomics profiling [24]–[27]. However, such molecular phenotypes are not routinely profiled in a typical high-throughput drug testing approaches, especially in clinical settings. Moreover, downstream changes in the expression patterns are shown to be suboptimal in distinguishing mechanism of action between different compounds [28], [29]. Perhaps more importantly, targets identified by means of genomic profiling may not be pharmaceutically actionable in clinical practice. For instance, many genes identified through expression profiling or genomic sequencing are either not druggable at all, or druggable, but not actionable, as there are no approved drugs available in the clinic.

In this article, we present an efficient model construction algorithm, named TIMMA (Target Inhibition inference using Maximization and Minimization Averaging), which makes the use of partly overlapping target subsets and supersets of promiscuous drug-target binding profiles in the estimation of anticancer efficacies for novel drug target combinations. The model construction and target combination predictions are based on functional data on drugs and their targets that are available from comprehensive target binding assays and from high-throughput drug sensitivity screens. We implemented a modified sequential forward floating search algorithm for model selection, which enables scaling-up to proteome-wide evaluation of the targets in terms of their relevance to cancer survival. Both simulation studies and an application to a canine osteosarcoma cell line data showed that TIMMA achieved improved prediction accuracy, when compared to a published algorithm [30], at significantly lower computational cost. Importantly, application case studies in MCF-7 and MDA-MB-231 breast cancer and BxPC-3 pancreatic cancer cells confirmed that TIMMA-predicted kinase targets are essential for tumor survival, either individually or in combination, as validated by independent single and pairwise target knockdowns with siRNA screening. Our model predictions, visualized as a target inhibition network, provide insights into such druggable cancer cell addictions, the inhibition of which can jointly block the survival pathways. With the increasing interest in drug combination screens, our modeling strategy can be readily used as an efficient prioritization procedure to pinpoint the most potential drug combinations based merely on their selectivity profiles and individual responses in given cancer samples.

## Materials and Methods

### A target inhibition model

Consider a set of 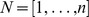 drugs where the single-drug treatment efficacy on a given cancer sample is measured as a phenotypic response in a high-throughput drug screen. The drug's treatment efficacy to kill cancer cells is conventionally scored using response parameters, such as the drug concentration at which the cancer cell growth is inhibited by a certain percentage (e.g. half-maximal inhibitory concentration IC_50_). A drug with a smaller inhibitory concentration is usually considered as more potent. Drug treatment efficacy and potency can be also quantified based on the area under the dose-response curve, such as the activity area (AA) [31] or the drug sensitivity score (DSS), which provide summary information about the complex dose-response relationships. We denote the drug treatment efficacy data by a vector 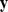 with length 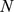 and scale it into the interval of [0, 1], with the minimum and maximum efficacies being 0 and 1, respectively.

To relate a drug's treatment efficacy with its underlying mechanism of action, the cellular targets of the drug need to be mapped into a drug-target inhibition profile. Let the potential target set be 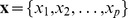, where 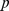 refers to the total number of targets that bind to at least one of the 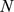 drugs. A target inhibition profile of a drug *i* can be binarized from drug-target binding affinities as a binary vector 
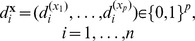
, where 0 and 1 is a result of classification of low and high binding affinities, respectively. The target inhibition profile for all the drugs is abbreviated as 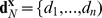. An example of such binarized target annotations can be derived from quantitative binding assay measurements collected from the ChEMBL database [32], provided that knowledge of relevant binding affinity cutoffs is applicable.

**Figure 1:**
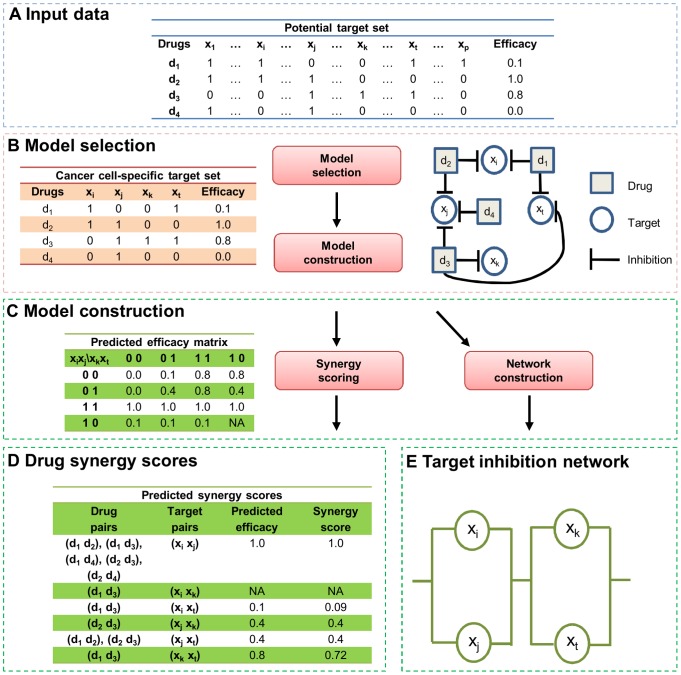
TIMMA model construction and prediction pipeline, with an illustrative toy example case. (**A**) The input data consist of the drug-target interaction profiles and the single-drug treatment efficacies. The targets that are inhibited by at least one drug in the given data matrix are considered as potential targets relevant for the survival of the particular cancer cell type. TIMMA first applies a model selection procedure to identify a panel of such targets that in combination explain the observed drug efficacies. (**B**) The identified subset of targets and the drug efficacy patterns in the given cancer type. In the next step, a model construction algorithm is applied on the reduced data matrix to predict the combinatorial efficacies of multiple target inhibitions. (**C**) The predicted efficacy matrix for the cancer cell-specific target set. (**D**) Based on the predicted efficacies, synergy scores are calculated for pairs of targets and the corresponding drug pairs. NA entries indicate those drug pairs that are non-identifiable by the model. (**E**) A visualization of the target inhibition network.

Given the single drug efficacy and target inhibition profiles, our aim is to predict the treatment efficacy for novel drug combinations. We consider the target inhibition profile of a drug combination as a union of the target inhibition profiles of each component drug in the cocktail (Figure 1). However, not all the targets in the profile are essential in explaining the treatment efficacy. Ideally, an effective drug combination should affect signaling pathways involved in cell proliferation and growth of the particular cancer type. In searching for a rational design in polypharmacology, one needs to first identify a set of targets whose interactions play critical roles in delivering the anticancer efficacy [9], [33]. Therefore, a fundamental computational problem is to identify a subset of therapeutic targets whose combinatorial interaction effects can be predicted in relation to cancer survival phenotypes. Note that in an individualized experimental setting, where different cancer types are tested for drug efficacy, the therapeutic targets should be also cancer-specific.

Let 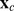 denote such a cancer-specific therapeutic target set. Identification of 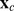 corresponds to a partition 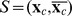 of the potential target set 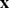 into two non-empty and non-overlapping groups. Let the space of distinct partitions for 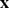 be denoted by 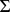. We will learn an optimal partition  from 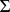 such that the cancer-specific targets can be separated from the remaining ones in 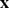. We assume that the drug target inhibition profiles 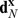 and the drug treatment efficacy data 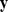 can be used for evaluation of target set relevance provided that 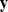 is a treatment outcome of drug perturbations on cancer survival pathways by multi-target inhibition in 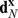. A plausible assumption is that the targets of more effective drugs are more likely to be involved in cancer survival pathways than those of less effective drugs. Therefore, targets that are predictive of drug efficacy are, in general, functionally important for cancer survival and should be selected with a higher probability for drug target combinations as well. More formally, the learning procedure for identifying such a cancer-specific target set 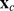 is to find a model that gives the best prediction performance. We are especially modeling multiple interactions among the target set 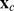 for the prediction of drug efficacies and therefore capturing the synergistic combination effects that cannot be revealed by inhibiting any of the targets individually.

Let 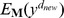 denote the model prediction error for a drug or drug combination 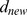 in a testing set. In its most basic form, the prediction error is calculated as the absolute difference between the predicted and the actual treatment efficacy:
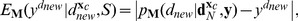
(1)where 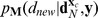 refers to the predicted efficacy for drug 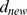 by a model 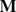 that takes 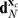 and 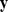 as training data. We take here a formal model-based strategy to estimate 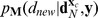 by formulating a predictive modeling framework for any training data 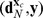; the model construction and model selection algorithms will be proposed in the sequel.

### TIMMA model for predicting drug efficacy

In an earlier work by Pal and Berlow [30], two fundamental set theoretic rules were exploited for predicting the drug efficacy according to its target profiles:

#### Rule on successful drugs

If a drug 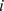 with target set 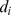 is successful to block cancer survival pathways, then a drug that inhibits a superset of 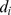 is also successful. That is: 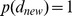 if 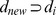 and 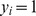.

#### Rule on unsuccessful drugs

If a drug 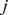 with target set 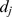 is unsuccessful to block cancer survival pathways, then a drug that inhibits a subset of 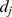 is also unsuccessful. That is: 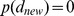 if 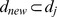 and 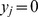.

The rationale for the two rules is straightforward. First, it is assumed that a drug inhibits cancer growth by switching off certain survival signaling pathways via modulation of its targets. Second, the topology of cancer survival pathways is perceived to be conserved irrespective of drug perturbations. These assumptions generally hold for multi-target inhibitors that are tested for treatment efficacy in a specific cancer subtype (Supplementary Figure S3). In contrast to conventional chemotherapeutic or cytotoxic drugs that lack cellular selectivity, signal transduction inhibitors more often target cancer-specific processes. The action of such targeted inhibitors, therefore, makes its therapeutic effect through blocking one or a few signaling pathways through its cancer-specific targets. Moreover, specific cancer cells under specific conditions, such as those in a cancer cell models, consist of rather homogeneous genetic make-up. The drug response data is usually profiled in a relatively short time scale, and it is therefore unlikely that there will be significant drug-induced topological changes in the cancer survival pathways.

Like with any modeling study, the prediction of novel drug combination efficacies based on the above assumptions simplifies the complex interactions between drug compounds and cancer phenotypes. For instance, some targets may interact with both successful and unsuccessful drugs due to protein promiscuity in ligand binding. To cope with such uncertainties that arise from experimental data, Pal and Berlow [30] adopted a stochastic extension to the two basic rules by taking an average of quantitative drug efficacy values, referred to as the Probabilistic Kinase Inhibition Map (PKIM) rule:
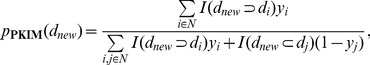
(2)Here 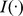 is an indicator function equal to one when the argument is true, and zero otherwise. The estimate Eq. 2 can be treated as the degree of treatment efficacy accumulated from the subsets of 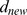, relative to the loss of efficacy in its supersets. Use of the indicator functions requires that both of the subsets and supersets of 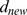 must be present in the data; otherwise PKIM will become non-determinable, as 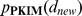 takes 0 or 1 irrespective of the actual profile of 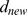. This non-identifiability problem due to data sparsity is a limiting factor of PKIM in many practical applications when scaling it up to the proteome-wide level, as the coverage and overlap between drug target profiles is often minimal under the high-throughput drug screen settings.

To address these limitations, we propose a TIMMA (Target Inhibition inference using Maximization and Minimization Averaging) model, which make the full use of the information content in the screening data to predict 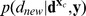. We consider sources of evidence that are determined from identical sets, subsets and supersets in 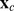 separately. Formally, the TIMMA model starts by taking the average of the efficacy values of those drugs whose target profiles are equal to 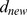:
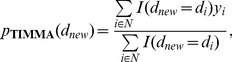
(3)where 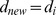 denotes that 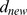 is identical to 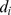. If no such an identical set exists, then TIMMA considers the following two prediction rules that are applied to the subsets and supersets separately.

#### Maximization prediction rule

For the subsets of 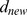 that can be found in 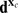, let the index for the drug that has the highest efficacy value be 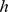, *i.e.* 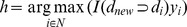. We initialize
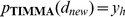
(4)If other drugs whose target profiles are subsets of 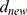 are also supersets of 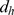, but their efficacy values are lower than that of 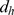, then Eq. 4 will be updated by taking the average:
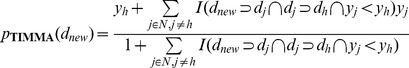
(5)


#### Minimization prediction rule

For the supersets of 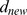, let the index for the drug that has the smallest efficacy value be 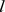, *i.e.* 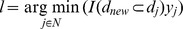. We initialize
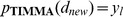
(6)If other drugs whose target profiles are supersets of 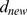 are also subsets of 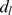, but their efficacy values are higher than that of 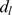, then Eq. 6 will be updated by taking the average:
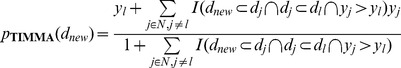
(7)The estimates in Eq. 5 and Eq. 7 can be interpreted as the lower bound and upper bound for 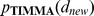. If both of these estimates can be learned from the data, then the average of them is taken as the maximum likelihood estimate for the predicted efficacy. The algorithm flow chart for TIMMA model construction is given in Supplementary Figure S1.

### Selection of cancer-specific targets using floating search

Construction of a TIMMA model for predicting drug efficacy requires a selection of cancer-specific target set 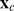 as the model parameter. Usually 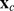 is *a priori* unknown and need to be inferred from the potential target set 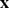. In our model-based learning framework, the likelihood of a proposed target set 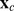 being composed of cancer-specific targets can be evaluated using the prediction accuracy of the corresponding TIMMA model that takes 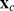 as its parameter. More formally, we consider an objective function for model selection as the average leave-one-out (LOO) TIMMA prediction error:
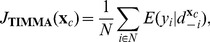
(8)where the leave-one-out prediction error 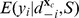 for drug 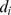 is given by Eq. 1 and Eq. 3–7. Given that the combinatorial space for 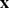 is huge for even a modest number of targets, it is not possible to calculate the objective function for all the possible target subsets 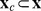 using exhaustive enumeration. We consider a Sequential forward floating search (SFFS) algorithm modified from [34] for minimizing Eq. 8 in a computationally efficient manner. The modified SFFS algorithm learns the optimal cancer-specific target set by aggregating and subtracting targets in 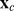 at different steps, as defined in the following, with the aim of minimizing the prediction error 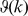, where 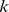 is the cardinality of set 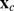,i.e.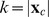:

#### Initialization

Evaluate the objective function Eq. 8 for single targets in 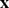. Determine the minimal prediction error 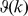 for 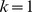 and select the corresponding target as the initial set of 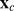.

#### Inclusion

For the current target set 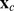 with 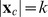, include a new target 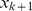 from the remaining set 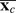 of 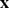, selected as the target the inclusion of which decreases the prediction error most. If no such a target exists in 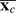, stop and exit the algorithm; otherwise update 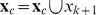 and 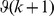 ; go to the Conditional exclusion step.

#### Conditional exclusion

After inclusion of 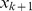, find in 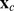 the target 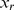 the removal of which leads to the minimal increase in the model prediction error. If 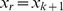 then keep 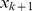 and go to the Inclusion step to include more targets; otherwise form a temporary set 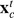 by excluding 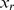 from 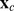. If 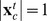, then update 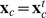 and go to the Inclusion step; otherwise go to the Continuation of conditional exclusion step.

#### Continuation of conditional exclusion

In the temporary set 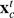, find the target 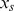 the removal of which leads to the minimal increase in the model prediction error. If the model prediction error for the reduced set 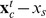 is smaller than the minimal error achieved in the previous iterations for the same size as 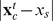, update 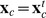 and then go to the Inclusion step; otherwise update 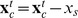 and repeat the Continuation of conditional exclusion step until no improvement can be made. If 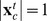 then update 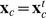 and go to the Inclusion step.

The sequential search strategy allows a dynamic change of the target set dimensionality and thus can overcome the limitations of many monotonic search algorithms, such as the greedy search in the PKIM algorithm [30], which have high tendency to get trapped to local optimal and may therefore fail to identify important target combinations. Importantly, the floating search algorithm does not require any *a priori* determined upper bound of 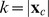 and it also enables a flexible exploration of target sets with high dimensions. The algorithm flow chart for TIMMA model selection is given in Supplementary Figure S2.

### Implementation issues to speed up computation

We have further improved the scalability of the TIMMA algorithm to large and complex data in MATLAB by exploiting its matrix computation architecture. Briefly, the TIMMA model was represented as a 3- dimensional array, where each drug's contribution to the estimate of 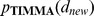 is calculated independently of each other. This multi-dimensional data structure has enhanced the computation efficiency significantly as most of the iteration loops can be avoided. Meanwhile, independent computing enables parallel distribution of the model prediction on separate processors, e.g. one processor for one drug, which will further decrease the computation time. For the SFFS target selection, the multi-dimensional data structure also facilitates the aggregation and comparison of prediction error 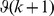 at the Inclusion step when the target 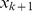 is added to 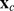, as 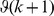 can be incrementally derived based on 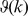 that has been obtained in the previous iterations. The TIMMA implementation code is freely available at http://timma.googlecode.com/.

### Scoring of synergistic drug pairs

For the optimal target set 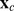 selected by the SFFS algorithm, the result of the TIMMA model prediction is summarized in the predicted efficacy matrix, which enumerates the treatment efficacy for each of the combinatorial target inhibition in 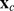 (Figure 1C). Here, we considered the predictions for the single and pairwise target inhibitions only, and derived a synergy score for the target pair (*A, B*) based on the multiplicative null model:
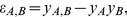
(9)where 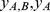 and 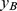 denote the predicted efficacies for the target pair and its individual targets, respectively. The multiplicative model is widely being used in the gene knock-out studies in model organisms to score quantitative genetic interactions between gene deletions [35], [36]. It has also been recently applied to investigate genetic interactions in human cancer cells using combinatorial RNAi screening [37], as well as to characterize drug synergy effects according to the Bliss independence model [38], [39].

Using the model predictions, we can calculate the synergy score also for those drug pairs (*d_1_, d_2_*) whose targets are included in 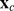. If one or both of the drugs are inhibiting multiple targets, e.g. 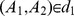 and 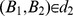, then we assign a drug synergy score for the drug pair using the mean of its corresponding target pair synergy scores defined by the multiplicative model (Eq. 9), i.e.
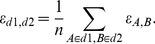
(10)In the given example, 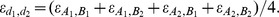 A deviation of 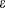 from zero provides evidence for a non-additive interaction between the two drugs, where 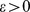 indicates synergy and 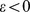 indicates antagonism.

### Scoring of synthetic lethal target pairs

When the target set size is fixed at two, the TIMMA model construction algorithm evaluates the pairwise target inhibitions without considering any higher-order interactions. This enables the TIMMA modeling strategy to systematically predict target pairs with synthetic lethality effect. By definition, synthetic lethality among a target pair states that: (i) inhibition of either of the single targets will result in incomplete cancer killing; and (ii) inhibition of both of the targets simultaneously will block the complete cancer survival sub-network. Therefore, the target inhibition network for the synthetic lethal target pair can ideally be represented as two nodes in parallel, similar to the topology of 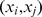 or 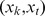 shown in Figure 1E. In comparison, there are two competing models: one with no links connecting the target nodes (referred to as a singleton model), and the other with two nodes linked in a sequence (referred to as a series model). Under the series model, no synthetic lethality effect is expected since the inhibition of a single target is already sufficient to block the cancer survival pathway. Therefore, from the model fitness perspective, we are expecting higher prediction accuracy for a synthetic lethal target pair under the parallel model, compared to both the series model as well as the singleton model. To evaluate the likelihood of a parallel model against the competing models for a given target pair (*A*, *B*), we defined a synthetic lethality score as the ratio of the fitness function of these two models, given by the total sum of squares (TSS) of the predictions:
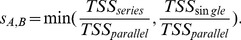
(11)The synthetic lethality score is conceptually different from the multiplicative synergy score as they are addressing different questions. The synthetic lethality score evaluates the pairwise target interactions by comparing the likelihood of three competing model structures, whereas the synergy score is derived based on the model averaging by combining all the possible models. Synthetic lethality corresponds to a special case of synergy, which requires minimal individual effects that are not considered explicitly in the multiplicative synergy score. Further, the higher-order target interactions, which are evaluated during the sequential forward search for the TIMMA model, are not considered when calculating the pairwise synthetic lethality score.

### Data sets

#### Simulated data

To initially benchmark the model performance, we generated simulated drug target data containing 100 drugs and 10 targets. The binding affinity values for the drug-target pairs were uniformly distributed in [0, 1], reflecting the most challenging case where the uncertainty about the drug-target interactions is maximal. The current implementation of TIMMA assumes binary drug target profiles so that the maximization and minimization rules can be directly applied on their subsets and supersets. For the simulated data, we evaluated the model performance on the full scale of binarized drug-target binding sets, generated by gradually increasing the binding affinity threshold with a step of 0.01 from 0 to 1. The drug treatment efficacy data 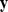 were uniformly sampled from [0, 1], where a high value implies a strong treatment effect on inhibiting cancer cell survival. We compared the performance of TIMMA and PKIM algorithms using the LOO prediction error in (8) on the binarized drug target data at varying thresholds. The computation times for TIMMA and PKIM were compared on the same dataset, keeping the number of targets fixed.

#### The CanOS1224 cell line data

We next applied TIMMA to the real drug sensitivity data used for evaluation of the PKIM algorithm [30]. Treatment efficacies were measured for 36 kinase inhibitors in a canine osteosarcoma cell line CanOS1224 by means of scaled IC_50_ values (positive values indicate increased efficacies for 12 compounds, while zero value means no efficacy). The kinase targets for these 36 targeted drugs were obtained from a comprehensive competition binding assay [40], where the binding affinities between these drugs and a selection of 317 kinases were measured by quantitative dissociation constant (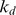) values (Dataset S1). The 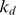 values were inversely scaled as 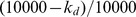 with the maximal concentration of 10000 nM, so that a high scaled 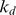 value implies a high binding affinity. A threshold of 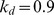 was considered in [30] to binarize the drug target data. However, as many of the weaker drug-target interactions cannot be ignored when defining the drug actions on cancer-specific targets [9], we assessed the models performance on the drug-target data obtained at lower cut-off thresholds as well.

#### The MCF-7 and BxPC-3 cell line data

To further demonstrate the model performance, we applied the TIMMA model to more challenging case studies in MCF-7 breast cancer and BxPC-3 pancreatic cancer cell lines. We utilized the recently published Cancer Cell Line Encyclopedia (CCLE) collection [31], which includes treatment responses in 479 human cancer cell lines to 24 anticancer drugs. In the CCLE data, the drug treatment efficacy was measured as the activity area (AA) under the dose-response curve to capture simultaneously the efficacy and potency of a drug. The AA scores were scaled to the interval [0, 1] for each of the cancer cell lines selected for analysis (Dataset S3). We found quantitative target specificity data (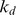 values) for 12 of the 24 drugs from a recent large-scale binding affinity mapping [41], where 72 kinase inhibitors were tested with 442 kinases covering the majority of the human catalytic protein kinome. Among the 442 kinases, we focused on catalytically active human protein kinases and thus removed kinases from human pathogens, as well as mutant kinases and noncatalytic kinases, resulting in a total of 384 kinases. From the 384 kinases, we excluded those targets that are inhibited by less than 2 of the 15 drugs, as these targets are lacking of drug efficacy information for estimation of their interactions. To obtain binary drug-target profiles, a drug-specific threshold (50-fold of the minimal 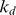 value for the particular drug) was applied. Similar drug-specific threshold was also used in [42] to define target binding classes. Further, we used binary target databases, such as TTD [43] and PubChem [44], to obtain target information for 3 additional kinase inhibitors (PD 0332991, saracatinib and PD-0325901). The remaining 9 drugs are not targeted kinase inhibitors and therefore were discarded in the following analysis (Dataset S4).

#### MDA-MB-231 cell line data

We considered 41 kinase inhibitors for MDA-MB-231 cancer cell line, which belongs to the triple-negative breast cancer subtype (TNBC). The drug-target interaction data was again retrieved from the kinome-wide binding affinity assay as reported in [41], where the same set of 384 kinases was selected for the TIMMA model predictions. The drug efficacy was quantified using so-called drug sensitivity score (DSS). Similarly to the activity area (AA) score utilized in the CCLE study [31], DSS summarizes the area under the dose-response function calculated by analytic integration over the concentration range. To favor on-target responses over toxic off-target responses, the integrated response was further normalized by the logarithm of the bottom asymptote (Dataset S7). We note that an optimal target set identified by TIMMA model may include targets that are already individually important for cancer survival, such as those that are involved in cell cycle arresting and apoptosis. Combination of these targets with others is expected to achieve a higher efficacy but provides a limited predictive power for the validation purpose. Therefore, we applied in this case study a more stringent validation procedure to test whether TIMMA can identify the synergistic effects among individually non-essential targets. Namely, we chose to exclude thee already known individually important targets, and experimentally validated only the ‘unexpected’ target combinations that are predicted by TIMMA. For this purpose, we carried out single and pairwise siRNA screens to knock-down targets and to compare their individual and combinatorial effects on inhibition of cancer cell viability.

#### Single and pairwise siRNA screens

siRNA against the selected genes and the positive and negative controls were purchased from Qiagen. Three different siRNAs combined against each gene were used. The final concentration of total siRNA was 6 nM for single gene knock-down. For double gene knock-down, the concentration of siRNA against each gene was 3 nM. The siRNAs were transferred on clear bottom 384-well plates (Corning #3712) using an Echo 550 acoustic dispenser (Labcyte). Lipofectamine RNAiMAX Transfection Reagent (Life Technologies) diluted according to manufacturers' instructions. In OptiMEM (Life Technologies) was added using a Multidrop Combi nl dispenser (Thermo Scientific). The plate was incubated for 1 h at room temperature in a shaker after which MDA-MB-231 cells (ATCC, cultured according to the provider's instructions) were added using a Multidrop Combi dispenser (Thermo Scientific). After 96 h incubation at 37°C, in 5% CO_2_ in a humidified incubator, cell viability was recorded by adding the Cell Titer Blue (Promega) reagent according to manufacturers' instructions and reading fluorescence at 595 nm using Pherastar FS (BMG) plate reader. The data was analysed using Dotmatics software (Dotmatics Ltd). Each plate was normalized against the positive and negative controls and the Z′-factors calculated were used to control the quality of each data set. Percent inhibition was then calculated for each siRNA combination, normalized against the positive and negative controls.

Quality control of the siRNA screen was done first by checking the Robust Z′-factor [45] for the raw intensities. The Robust Z′ factor was 0.71, passing the quality assessment threshold (0.5) with a sufficient difference between background noise and true signal [46]. Reliability of the cell inhibition percentages was further assessed by the correlation between two technical replicates. The overall rank correlation is 0.896 (p<10^−15^), indicating a consistent readout for the same double gene knock-downs. Therefore, the inhibition percentages were averaged for each double gene knock-down, except for those where the two replicated cell inhibition percentages differ more than 15%, in which case the replicate that is located at the edge of the plate was excluded. Ten replicates (3% of the total data) were removed due to such edge effects. To make the single and double gene knock-down results more comparable, the single gene knockdown effects were normalized by taking the average of the inhibition values of single gene knock-down and those double gene knock-down that include this gene with lower inhibition values (Dataset S8). The multiplicative synergy scores for the siRNA target knock-downs were calculated the same way as for the TIMMA predictions, with 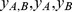 indicating the measured inhibition percentages of the cell viability for pairwise and single siRNAs, respectively, using Eq. 9. The synergy score for the drug pairs was derived similarly as for the TIMMA predicted drug efficacies using Eq. 10.

## Results

To evaluate the relative efficiency and accuracy of TIMMA, we initially compared the TIMMA and PKIM algorithms on the simulated data and on the CanOS1224 canine osteosarcoma cell line. In the more practical case studies, we then applied the optimized TIMMA model to infer effective drug targets in the context of MCF7 breast cancer and BxPC3 pancreatic cancer cell lines, where kinome-wide siRNA knockdown data are publicly available for experimental validation. Finally, we evaluated the synergistic effects of the predicted drug target combinations in the MDA-MB-231 breast cancer cells by carrying out pairwise siRNA silencing screens for the TIMMA-selected kinase targets.

### Model performance on the simulated data

**Figure 2:**
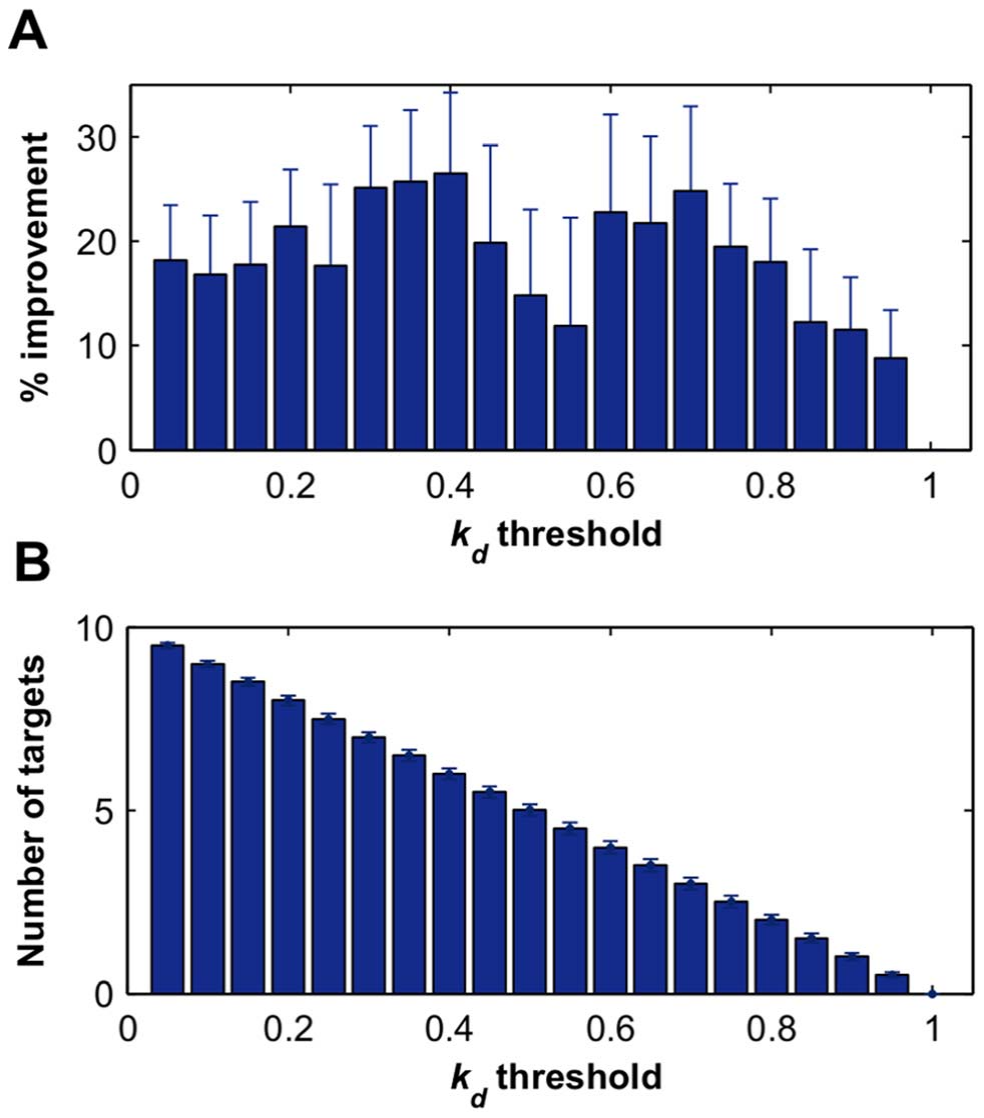
Prediction accuracy and drug promiscuity as a function of binding affinity threshold on simulated datasets. (**A**) The relative improvement of the LOO prediction accuracy when comparing the TIMMA and PKIM models. The 95% confidence interval for the average percentage improvement relative to PKIM was derived empirically by repeating the data simulation 100 times. (**B**) The average number of targets per drug and its standard deviation interval when applying different cut-off 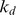 thresholds to binarize the simulated binding affinity data.

We started by evaluating the relative performance of TIMMA and PKIM in terms of their accuracy in predicting the treatment efficacies for new drugs on the simulated dataset. It was found out that TIMMA systematically improved the average leave-one-out (LOO) prediction accuracy, compared to PKIM, at each predefined drug-target threshold (Figure 2A, paired t-test, p = 5.0024×10^−10^). Since TIMMA combines the information from a drug's subsets and supersets simultaneously, its predictions are more robust to data noise and other technical factors that are inconsistent with the model assumptions, compared to PKIM, which does not consider model averaging. In particular, TIMMA gains on average 22.4% increase in the prediction accuracy especially for affinity thresholds lower than 0.8, which correspond to the promiscuous cases with, on average, more than two targets per drug (Figure 2B). These results demonstrate the importance of the improvements provided by the TIMMA algorithm, which make it applicable also to more challenging and practical cases, where target promiscuity is common and knowledge about all the cellular targets of drugs is rarely available.

**Figure 3:**
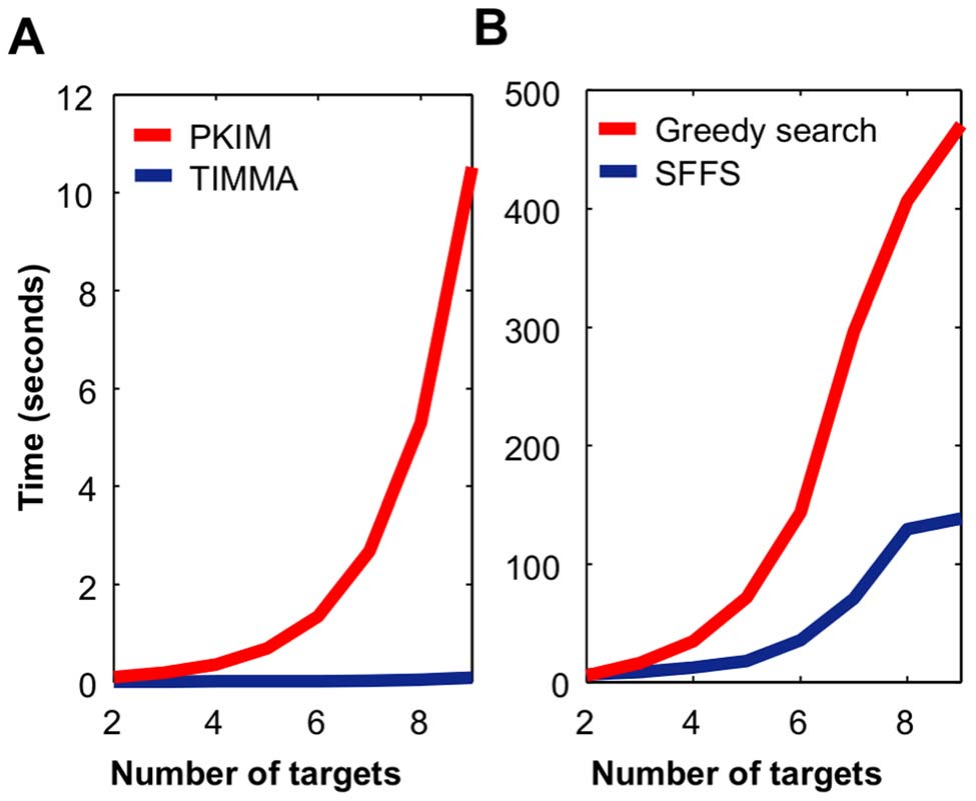
Computation times on simulated datasets with a varying number of targets. (**A**) Running time of PKIM and TIMMA model construction algorithms given a target set that contains 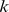 targets, 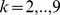. (**B**) Running time of the greedy search and the sequential forward floating search (SFFS) algorithms when reaching an optimal cancer-specific target set of size 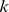.

Another important consideration in the large-scale drug screens is the computational complexity of the prediction algorithms. The computation times for TIMMA and PKIM model construction algorithms, SFFS and greedy search, respectively, were compared on a standard 2.6 GHz desktop computer. In contrast to the exponentially increasing time that is needed for the PKIM model construction, TIMMA takes approximately linear increase in time with the number of targets (Figure 3A). Even though the SFFS is computationally more demanding than greedy search in model selection, TIMMA achieved marked speed-up due to the optimization techniques using multi-dimensional matrix computations (Figure 3B). Notably, with 20 targets and 10 drugs, for example, the greedy search will take 10 days, while the TIMMA takes on average 30 minutes to complete, and thus saves up 99% of the computation time. The enhancement in the computation speed facilitates the analyses of larger and more complex datasets with increasing number of drugs and their target information.

### Model performance on the CanOS1224 cell line data

**Figure 4:**
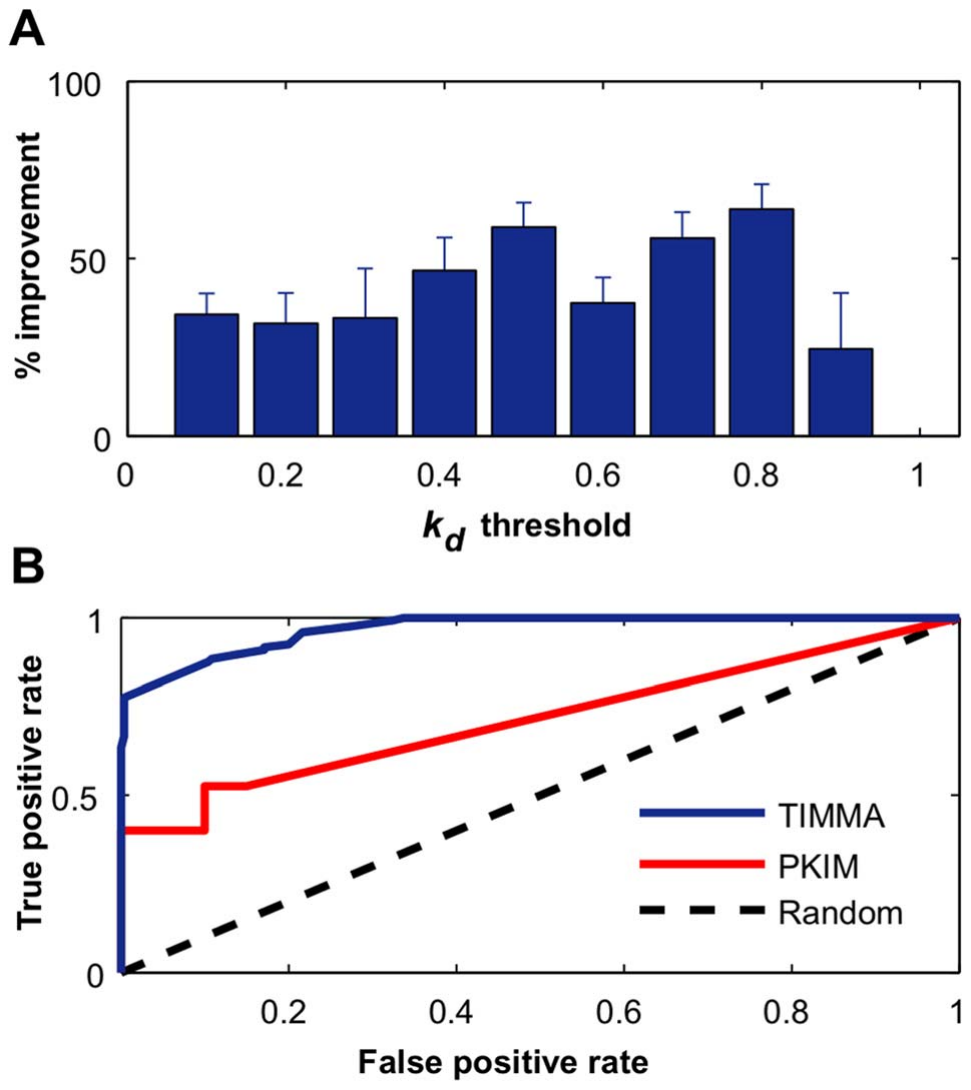
Prediction accuracy of TIMMA and PKIM on the CanOS1224 data. (**A**) Relative increase of average LOO prediction accuracy for TIMMA compared to PKIM. The drug-target data was binarized at various binding affinity thresholds, ranging from 0 to 1, with a step of 0.01. For each of the binarized drug-target data, the optimal cancer-cell specific target sets identified by TIMMA and PKIM were compared in terms of prediction accuracy. The 95% confidence interval for the relative increase was derived empirically using 50 random starting points in the model selection algorithms. (**B**) The receiver operating characteristic (ROC) curves for classifying sensitive drugs. Model predictions given the binarized drug-target data were pooled together for evaluation of the classification performance on the set of 36 drugs, where drugs with positive scaled IC50 efficacies are labeled as sensitive.

We next tested whether TIMMA can lead to improvements in the real dataset used in the PKIM work [25], first by fixing the 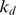 threshold at 0.9. From the set of 317 kinases, the PKIM model identified 8 kinases with a mean LOO error of 0.1314, while TIMMA identified a different set of 8 kinases with a decreased LOO error of 0.0574 (Dataset S2). When varying the 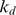 threshold, the average LOO prediction accuracy of TIMMA was significantly better than that of PKIM (Figure 4A, paired t-test, p = 1.3910×10^−5^). Similarly as in simulated dataset (Figure 2A), the improvement in the prediction accuracy varied with the selected cut-off threshold (Figure 4A). As expected, when the threshold is close to 1, the two models performed equally well, as the drug-target information is too few to make any reliable predictions; while TIMMA again systematically outperformed PKIM at the smaller thresholds.

As revealed in many kinome-wide drug binding assays, most drugs, albeit considered previously specific to single or double targets, have shown a relatively wide range of binding affinities to multiple off-target kinases [47]. Our model can also make use of such promiscuous drug-target interactions that are informative for predicting drug cancer killing efficacies. This was further investigated in a receiver operating characteristics (ROC) analysis of the prediction performance, where the problem was to distinguish the 12 most sensitive drugs with positive efficacy values (Figure 4B). In this analysis, the area under the ROC curve (AUC) for TIMMA was 0.9679 and for PKIM 0.7144, further demonstrating the improved predictive power of the TIMMA model.

**Figure 5:**
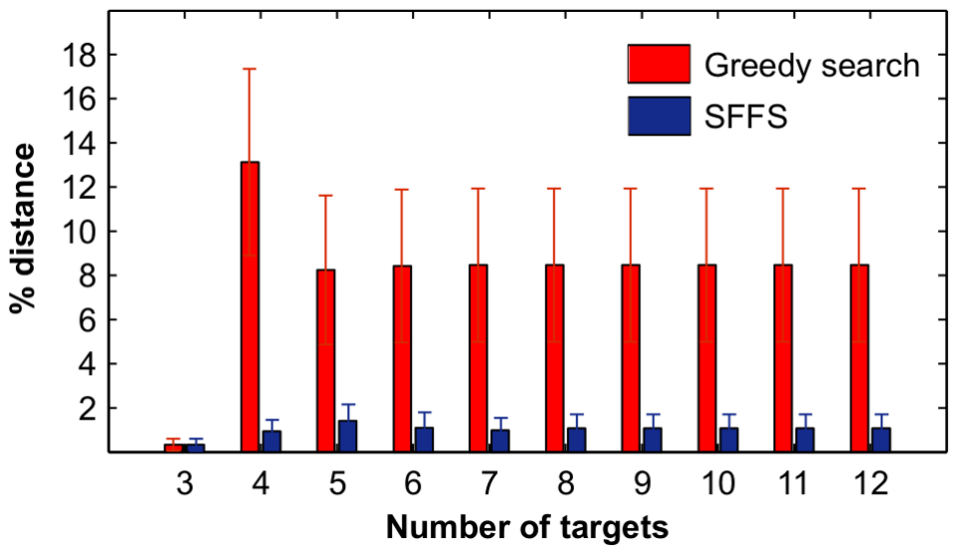
Optimality of the model search algorithms as compared to the global optima on the CanOS1224 data. The optimality of a search algorithm was evaluated by the relative distance in average LOO prediction error between the algorithm solution and the global optimum determined by an exhaustive search. The 95% confidence intervals were derived based on a 100 times sampling of *k* kinases from the 317 kinases, *k* = 2,…,12.

To test whether the SFFS model selection algorithm can find solutions close to the global optimal target sets, we performed an exact analysis for maximally 12 kinases, where exhaustive search can be performed at a reasonable running time. More specifically, 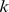 kinases from the full set of 317 kinases were randomly selected, where 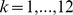, and an exhaustive search was run to determine the optimal subsets of the 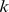 kinases. We applied here a fixed cut-off threshold of 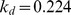, which equals to the average of all the 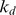 values over the drug-target pairs. The optimal sets determined by the SFFS algorithm in TIMMA and by the greedy search algorithm in PKIM were compared with the global optimum in terms of prediction accuracy. The SFFS algorithm gave significantly better results than the greedy search for 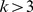 (Figure 5, paired t-test, p = 3.3397×10^−6^). This demonstrates that the computationally efficient SFFS algorithm can find solutions that are not too far from the globally optimal solution.

### MCF-7 breast cancer cell application

After confirming the appropriate performance of the TIMMA model, we applied it to two practical case studies. In the first one, we systematically evaluated the predictions of the TIMMA MCF-7 model against the experimental results from an independent kinome-wide siRNA study in the MCF-7 breast cancer cells [48]. The knock-down data were generated using a Methylene blue assays to assess cancer cell density in order to evaluate the quality of their siRNA screen (Figure S2 and Table S2 in [48]). The siRNA screen was designed to target 712 kinases in the human kinome, with three distinct siRNAs per kinase. The data was analyzed using the R package cellHTS2 [49], where a mean Z-score scaled by the per-plate median of the intensities of the negative controls was calculated for each kinase. A large positive Z-score indicates a strong inhibition effect and thus indicates high essentiality of the kinase for the cancer cell survival.

**Figure 6:**
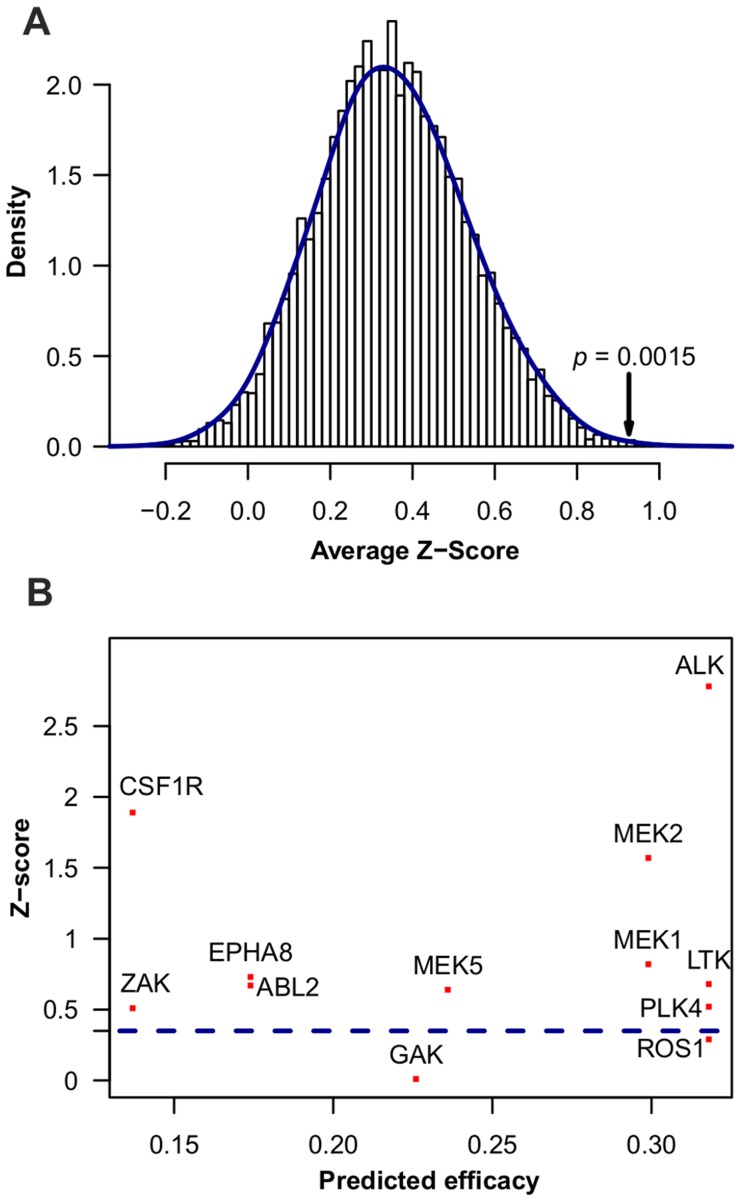
Kinases selected by TIMMA on the MCF7 cancer cell line. (**A**) Histogram of average siRNA Z-scores for a set of 12 kinases selected randomly, as compared to the average Z-score (0.926) for the TIMMA-selected optimal target set (marked on the right tail with its empirical *p*-value). (**B**) Scatter plot between the predicted treatment efficacy and the siRNA Z-score for the selected 12 kinases. The average Z-score (0.349) for the kinome-wide siRNA data is plotted as the dotted horizontal line.

Here, we tested the essentiality of the kinases in the cancer-specific target set predicted by TIMMA using the 15 drugs targeting a total of 384 kinases. In other words, we asked the question: are the kinases selected by TIMMA as the most predictive of anticancer efficacy also highly essential individually for the cancer cell viability? The optimal target set found by TIMMA included 12 kinases {ZAK, CSF1R, GAK, MEK5, ABL2/EPHA8, ALK/LTK/PLK4/ROS1 and MEK1/MEK2}, with a mean LOO prediction error of 0.1392 (Dataset S5). The/symbol stands for the targets that are inhibited by the same set of drugs in the data and thus are indistinguishable by the model. The mean Z-score for these 12 kinases was 0.926, which is significantly higher than the average Z-score for random sets of 9 kinases selected from the 712 kinases (Figure 6A, permutation test, p = 0.0015). This shows that TIMMA tends to choose, in general, such kinases that are also individually more effective in blocking cancer cell growth. Among these kinases, ALK had the highest predicted single-kinase efficacy. ALK was also identified in the independent siRNA screen as the top essential kinase. However, our model does not assume that all the kinases in the optimal target set are essential individually. For instance, GAK and ROS1 had a relatively low Z-score, but still these were considered to have an important role in the cancer survival and/or proliferation process when combined with the other selected kinases (Figure 6B).

On the basis of the predicted efficacy matrix based on the selected kinase targets (Dataset S5), we derived the multiplicative synergy score (Eq. 9) for the drug pairs that are pairwise inhibiting the selected targets (Supplementary Table S1). We found that the top synergistic drug pairs are mainly GAK and ALK/LTK/PLK4/ROS1 inhibitors, some of which have been reported in the recent literature. For example, crizotinib combined with erlotinib has recently been shown to cause a complete and genotype-specific inhibition of tumor growth in non-small cell lung cancer (NSCLC) adenocarcinoma patient-derived pre-clinical treatment models *in vivo* [50]. Crizotinib-erlotinib combination was also ranked as the top one among the 12 drugs that are available in the MCF-7 model analysis, indicating that such a combination might also be effective for the treatment of specific resistive subtypes of breast cancer. Similarly, TAE-684, a potent ALK inhibitor has been found to provide selective activity against those mutations that conferred crizotinib resistance in cancer patients [51], suggesting a mechanistic insights into the crizotinib-TAE-684 combination, which was ranked as the second most synergistic pair by our model predictions. In general, the top-predicted synergistic drug pairs are not necessarily the individually most sensitive drugs, as their individual efficacies do not correlate with the multiplicative synergy score (Supplementary Table S1).

**Figure 7:**
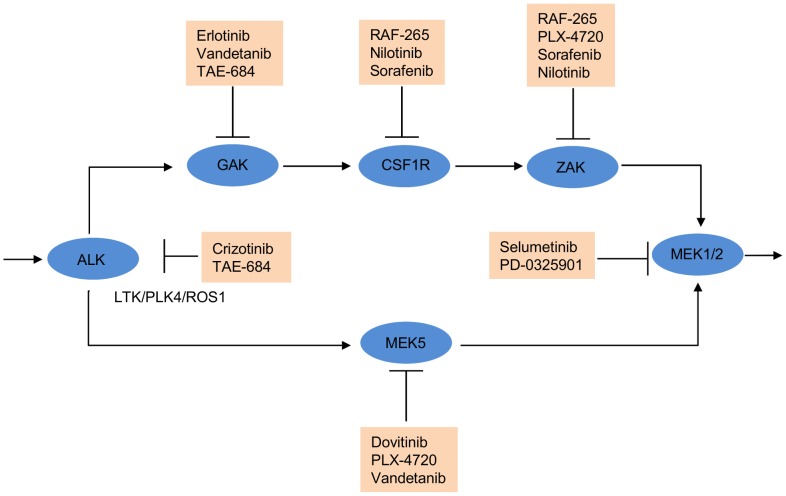
The MCF-7 breast cancer target inhibition network annotated with drugs inhibiting its target nodes. The target inhibition network was derived from the predicted efficacy matrix for the 12 kinase targets selected by TIMMA. Target pairs with predicted efficacy higher than 0.31 were considered as effective. Potential drug combinations can be inferred from the network by checking whether their targets are blocking the two parallel cancer survival pathways. Blue circle, target node; red square, available drugs that inhibit the target node; “/”, those targets that are inhibited by the same set of drugs and thus are indistinguishable by the model. The predicted efficacy matrix is provided in Dataset S5.

To visualize the combinatorial effect of the selected kinase targets, a target inhibition network was constructed by applying a threshold of 0.318 to binarize the predicted efficacy (Figure 7, Dataset S5). The threshold 0.318 was the scaled drug efficacy for crizotinib that inhibits ALK, which is the most essential kinase according to the siRNA screen and thus considered as effective in treating MCF7 cancer cells. The target inhibition network suggested that two parallel MEK1/2-dependent pathways as most important for the MCF-7 cancer cell survival. For example, simultaneous targeting of CSF1R and ALK/LTK/PLK4/ROS1 was predicted to enable blocking the two redundant pathways and result in a complete inhibition of the MEK1/2-dependent cell proliferation. Notably, CSF1R has been shown to act upstream of MEK1 and to induce Cyclin D2 expression via the Ras/Raf/MAPK pathway [52]. Similarly, ALK has been suggested to directly activate MEK1/2, independent of c-Raf [53]. Also, LTK has been implicated in cell growth via MAPK signaling [54]. Taken together, these findings support the idea that inhibition of both CSF1R and ALK/LTK/PLK4/ROS1 should have a synergistic effect on the cell survival. Indeed, the combination of sorafenib and crizotinib, inhibitors of CSF1R and ALK/LTK, respectively, has been considered for a clinical trial for treating advanced solid tumors (Pfizer, ClinicalTrials.gov, Identifier: NCT01441388).

### BxPC-3 pancreatic cancer cell application

To further show the applicability of TIMMA to such cases where combinatorial effects of kinase inhibition are considered, we utilized the results from a kinome-wide drug sensitization screen, in which the kinase siRNA-silencing was combined with the treatment of Aurora kinase inhibitors in BxPC-3 pancreatic cancer cell line [55]. Aurora kinases (Aurora A, Aurora B, and Aurora C) are serine/threonine kinases that are frequently overexpressed in many tumors. Accordingly, Aurora kinase inhibition has been proposed as potential cancer therapy to disrupt cancer cell division. The purpose of the study was to identify those kinases that when silenced would sensitize pancreatic cancer cells to the Aurora kinase inhibitor treatments. The RNAi screen was done using the Human Validated Kinase Set (HVKS) siRNA library from Qiagen, with two siRNAs per kinase. A total of 17 kinases were identified and confirmed in a validation screen to have at least 2 out of 4 siRNA sequences showing greater than 1.5-fold decreases in EC50 or EC30 values of the Aurora kinase inhibitor AKI-1 in dose-response curves [55].

We wanted to evaluate here the TIMMA model performance in predicting the experimental results in [56], especially the kinases that would sensitize the pancreatic cancer cells to the AKI-1 treatment. This question can be addressed in TIMMA by determining the synthetic lethality score for such kinases paired with the targets of AKI-1. The synthetic lethality score (Eq. 11) was calculated for the kinase pairs using the data of 15 drugs and 384 kinases and the drug efficacy in BxPC-3 cells [31]. The higher the score, the stronger the synthetic lethality effect for the kinase pair. Of these 15 drugs, 3 drugs (CHIR-265/RAF-265, nilotinib and PD0332991) were not tested for BxPC-3 and thus were removed (Dataset S3). Since none of the 12 compounds effectively targeted the two Aurora kinases, Aurora A and Aurora C, we considered here the Aurora B kinase as the only effective target of AKI-1. The TIMMA model was therefore tested on all those kinase pairs which contain Aurora B, and those kinase pairs whose synthetic lethality scores were higher than that of {Aurora B, Aurora B} pair were considered as synthetic lethal partners of Aurora B.

**Figure 8:**
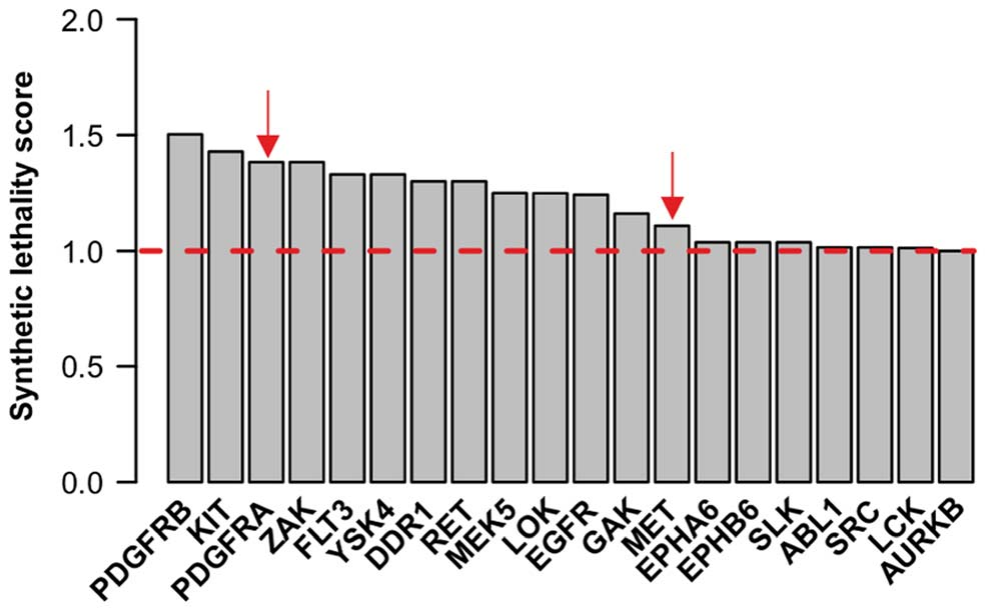
Kinase targets indicative of sensitizing BxPC-3 pancreatic cancer cells to Aurora kinase inhibitors. The TIMMA model identified 19 kinases that, when combined with AURKB, showed higher synthetic lethality scores than using AURKB alone. Among these targets, PDGFRA and MET (marked with red arrows) were experimentally validated in the original work [56]. Dashed line: the baseline synthetic lethality score when AURKB is combined with itself.

The TIMMA analysis based on Eq. 11 identified 19 kinases (multiple kinases are ranked the same as they are targeted by the same drug set), which showed stronger synthetic lethality interactions with Aurora B than with itself (Figure 8). Two (MET, PDGFRA) out of the three targets (MET, PDGFRA and PYK2) were experimentally validated as sensitizing targets of AKI-1 in the pancreatic cancer, representing a highly significant enrichment (hypergeometric test, p = 0.0046) (Figure S4 in [55]). In addition, the model predicted that PDGFRB might also be a potential sensitizer of AKI-1 treatment. Similar to the result in the MCF-7 cells, ZAK (ranked 3^rd^), MEK5 (ranked 7^th^) and GAK (ranked 9 ^th^) were again found in the cancer-specific target set for BxPC-3 cells, suggesting that the synergy patterns of these kinases is common across these cancer types. In contrast, the model predicted that the combination of MEK1/MEK2 and AURKB inhibitors has least synthetic lethal capacity (Dataset S6), because individual essentiality of these two factors favors the series connection model rather than the parallel model in the synthetic lethality score [56], [57].

### MDA-MB-231 breast carcinoma application

**Figure 9:**
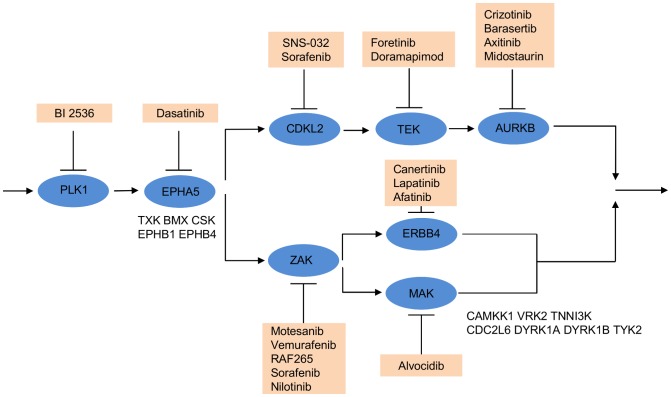
The MDA-MB-231 breast cancer target inhibition network annotated with inhibiting drugs. Blue circles represent kinase target nodes. A target node may contain multiple kinases that are inhibited by the same set of drugs; in such cases, the kinase with the minimal binding affinity 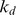 is shown inside the node, while the other equivalent kinase targets are shown beside the meta-target node. Red squares list available drugs that inhibit the corresponding target nodes. Data and detailed results are provided in Dataset S7.

The final application case study was the human triple-negative breast carcinoma, where we experimentally validated the TIMMA target combination predictions using single and pairwise siRNA knock-downs on the MDA-MB-231 cells. The TIMMA model selected 20 optimal kinase targets {PLK1, AURKB, CDKL2, ZAK, ERBB4, TEK, TXT/BMX/CSK/EPHA5/EPHB1/EPHB4, CAMKK1/MAK/VRK2/TNNI3K/CDC2L6/DYRK1B/DYRK1A/TYK2} with an average LOO error of 0.11 (Dataset S7). These kinases and their functional interactions were mapped to the target inhibition network, which contained a total of 8 target nodes (Figure 9). The kinases belonging to the same node are inhibited by a common set of drugs, and therefore these drug targets are indistinguishable in terms of drug inhibition and their predicted efficacy values. Two of the selected kinase targets, PLK1 and AURKB, are known to be essential for cell growth, therefore serving here as positive controls for the model target predictions. However, due to their known role in cell growth, we excluded these two kinases from the experimental evaluation, and focused on the synergistic combinations between the remaining 18 kinases targets among the 6 target nodes.

**Figure 10:**
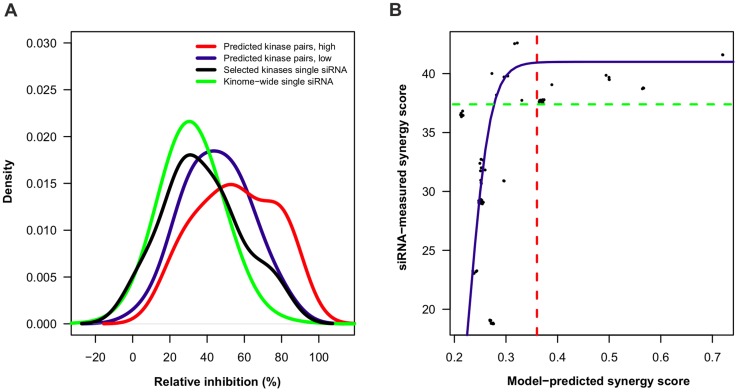
Experimental validation of the model predictions on the MDA-MB-231 cell line. (**A**) Distributions of cell growth inhibition percentages in single and pairwise siRNA screens targeting different groups of kinase targets. TIMMA identified eight target nodes, with a total of 20 kinase targets, the essentiality of which was evaluated both individually (n = 18) as well as in combination (n = 153) in the siRNA screens. Kinase target pairs with the predicted efficacy values higher or lower than the average (0.594) were further classified as high or low sensitivity groups, respectively. The kinome-wide single siRNA screen included a total of 704 kinases as a background reference distribution. (**B**) Scatterplot of synergy scores derived from the TIMMA model prediction versus synergy scores derived from the pairwise siRNA screen for a total of 68 drug pairs. A jitter function was applied for distinguishing the different drug pairs having the same synergy scores. The red dashed line indicates an empirical cut-off of predicted synergy score of 0.36 for the classification of highly synergistic drug pairs, for which the corresponding siRNA-measured synergy scores are higher than 37% (green dashed line). The blue curve is the logistic growth function fit 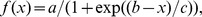 where *a* = 41, *b* = 0.23, *c* = 0.02.

In general, there were significant differences between the TIMMA-selected kinase targets, when these were silenced either individually or in combination in the siRNA screens, especially after their ranking according to the predicted efficacy (Figure 10A, Kruskall-Wallis rank sum test, p<10^−15^). Even after excluding the two essential kinases (PLK1 and AURKB), the 18 TIMMA-selected kinases showed higher cancer cell growth inhibition power in the single knock-down experiments (22% increase in cell inhibition), compared to the inhibition observed in the kinome-wide single-siRNA screen (Wilcoxon rank sum test, p = 0.28, Supplementary Table S2). Importantly, the 153 TIMMA-selected kinase pairs resulted in highly significant cancer cell killing improvement in the pairwise knock-down experiments (38% increase), compared to their single kinase inhibition efficacy (p = 0.0089, Bonferroni adjustment), indicating that TIMMA could select such kinase targets that, in general, are important for cancer cell survival, and especially when combined. Notably, when categorizing the selected target pairs as High and Low efficacy groups, according to their predicted treatment efficacies above or below the average of 0.6, there was a significant increase in the cancer cell growth inhibition percentages (23%, 48% and 80%), when comparing the High efficacy group to either the Low efficacy group, the single selected kinases or the kinome-wide background (p = 0.031, p = 0.013, p<10^−15^, Bonferroni adjustment, Supplementary Table S2). Taken together, these results indicate that the TIMMA model can effectively select and prioritize among the massive number of possible combinations those target combinations that are most potential for experimental testing or eventual clinical translation.

To investigate whether the model can select also such drug target combinations that individually show relatively low drugs efficacies, but will lead to increased drug synergy when combined, we focused on the set of 15 kinase pairs among the 6 target nodes ({CDKL2, ZAK, ERBB4, TEK, TXT/BMX/CSK/EPHA5/EPHB1/EPHB4, CAMKK1/MAK/VRK2/TNNI3K/CDC2L6/DYRK1B/DYRK1A/TYK2}, Figure 9) that are unique in terms of their drug profiles and thus distinguishable based on their TIMMA-predicted efficacy. We took an average of the synergy scores for those kinas pairs that are represented by the same target node pair. The synergy score calculated on the basis of the TIMMA-predictions correlated significantly with the synergy calculated on the basis of the single and pairwise siRNA measurements (Kendall correlation 0.39, p = 0.0463). When mapping the selected kinase target pairs to the available kinase inhibitor pairs, i.e. using Eq. 10, the correlation between the predicted and measured synergies improved further (Figure 10B, p = 0.0002). In particular, when using a cut-off predicted synergy of 0.36 (the dotted vertical line), the likelihood of obtaining a high measured synergy increased significantly (Wilcoxon rank sum test, p<5.9^−7^, Bonferroni adjustment). Among these top-20 most synergistic drug combinations for the MDA-MB-231 cells, there were a number of examples, such as the two top pairs, where the efficacy of one of the drugs in the combination was relatively low, or even zero, yet the predicted and measured synergy for the drug combination was high (Table 1). This demonstrates that our model is able to predict not only those pairs that are essential either individually or in combination, but also a number of synergistic combinations, where the predicted efficacy cannot be explained by the efficacy of the two single compounds when used alone (Supplementary Figure S4).

**Table 1:** The top most synergistic drug pairs based on the TIMMA model predictions in MDA-MB-231 breast cancer cells.

	Individual efficacies		TIMMA prediction		siRNA knock-down	
Drug pair	DSS_1_	DSS_2_	Predicted efficacy	Synergy score	Inhibition percentage	Synergy score
**Dasatinib Doramapimod**	9.05	0	0.723	0.723	63.399	41.588
**Dasatinib Foretinib**	9.05	0	0.723	0.723	63.399	41.588
**Afatinib Dasatinib**	9.05	1.73	0.723	0.569	54.822	38.607
**Canertinib Dasatinib**	9.05	1.93	0.723	0.569	54.822	38.607
**Dasatinib Lapatinib**	9.05	4.34	0.723	0.569	54.822	38.607
**Dasatinib Motesanib**	9.05	7.75	0.723	0.496	42.47	39.622
**Dasatinib Nilotinib**	9.05	4.22	0.723	0.496	42.47	39.622
**Dasatinib RAF265**	9.05	3.76	0.723	0.496	42.47	39.622
**Dasatinib Vemurafenib**	9.05	0	0.723	0.496	42.47	39.622
**Dasatinib Sorafenib**	9.05	7.03	0.723	0.388	50.299	39.022
**Motesanib SNS-032**	7.68	7.75	0.562	0.369	40.276	37.683
**Motesanib Sorafenib**	7.03	7.75	0.562	0.369	40.276	37.683
**Nilotinib SNS-032**	7.68	4.22	0.562	0.369	40.276	37.683
**Nilotinib Sorafenib**	7.03	4.22	0.562	0.369	40.276	37.683
**RAF265 SNS-032**	7.68	3.76	0.562	0.369	40.276	37.683
**RAF265 Sorafenib**	7.03	3.76	0.562	0.369	40.276	37.683
**SNS-032 Sorafenib**	7.68	7.03	0.562	0.369	40.276	37.683
**SNS-032 Vemurafenib**	7.68	0	0.562	0.369	40.276	37.683
**Sorafenib Vemurafenib**	7.03	0	0.562	0.369	40.276	37.683

DSS_1/2_, measured treatment efficacy (drug sensitivity score) when using either of the suggested drugs alone; Inhibition percentage, averaged inhibition of cell growth in the pairwise siRNA screen; Predicted efficacy, predicted treatment efficacy by the TIMMA model; Synergy score, synergistic effect of the drug pair as either measured by the siRNA experiment (Eq. 9) or predicted by the TIMMA model (Eq. 10). The table lists the drug pairs with synergy scores higher than the selected cut-off threshold (0.36, red dotted line in Figure 10B).

## Discussion

In this study, we utilized the principles of polypharmacological target inhibition modeling as a generic framework for pinpointing cancer-specific targets and predicting the effect of putative drug combinations. The main contribution of the present work was to introduce a novel model construction model, called TIMMA, and to demonstrate its feasibility in systematic investigation of the model predictions using kinome-wide single and pairwise siRNA knock-down experiments. We also showed that our enhanced model construction algorithm resulted in significantly better predictive accuracy and computational efficiency, compared with an existing algorithmic solution. With such improvements, the number of targets that can be included in the minimal set can go up to 20, which corresponds to maximally 20 drugs in a combination. In the three case studies, where we combined large-scale drug sensitivity screening and comprehensive drug-target data, we were able to identify a number of potential drug combinations for breast and pancreatic cancers. In more general terms, the optimized experimental-computational approach, empowered by the target inhibition network, allowed us to systematically explore how the kinase inhibitors and their cellular targets interact to modulate cancer growth phenotype on a global network-level, with the aim to identify molecular pathways behind drug action, as well as to suggest combinatorial treatment strategies that can block the cancer escape pathways and therefore tackle the resistance problem of the many current treatments approaches.

Network-based strategies, such as the one developed in the current work, provide a principled approach to systematically identify the key set of druggable vulnerabilities of cancer networks. Such efforts create a solid foundation towards implementing the emerging paradigm in drug discovery, the so-called ‘network pharmacology’ [3], which provides a more global understanding of the mechanism behind drug action and resistance by considering drugs and targets in their context of cellular networks and pathways. The current work also support the detection of synthetic lethal interactions, which is another conceptual framework recently proposed toward developing more effective therapeutic strategies [12], [13], [15]–[19]. More specifically, targeted perturbation or inhibition of a gene that has a synthetic lethal relationship with a driving cancer mutation holds great promise for being a highly specific and selective means to kill cancer cells without severe side-effects to normal cells. Compared to the conventional cytotoxic drugs, that affect both normal and cancerous cells, synthetic lethality can therefore address the fundamental challenges of anticancer therapy by optimally targeting differential features in each cancer type while sparing normal cells. However, despite the advances in siRNA and compound screening, synthetic lethal interactions between genes and/or drugs have remained extremely difficult to predict on a global scale [13], [18]. Network-based methods provide a convenient platform to finding functional interactions in disease networks, toward enabling identification of such effective drug targets and their combinations that tailored for more effective and personalized cancer medicine.

We focused here on the kinase targets because of their importance in many multi-target cancer treatment developments. This is also why we experimentally validated the model predictions using kinome-wide single siRNA and TIMMA-predicted pairwise siRNA screens, where the selected kinase targets were knocked down individually or in pairs in the given cell type to experimentally evaluate their essentiality either alone or in combination for the cancer cell survival. However, the same modeling principles could be applied also to other target families, such as enzymes or G protein coupled receptor (GPCR) targets, provided there will be enough target and drug promiscuity to allow for construction of the target inhibition networks. Moreover, while the siRNA silencing screens are convenient for the drug target investigation, the perturbation effects from the siRNAs cannot fully mimic the phenotypic effects of drug treatments. RNAi has also potential limitations due to potential off-target silencing effects and variable reagent efficacy, which may also partly explain the observed discrepancies between the drug treatment-based model predictions and their siRNA-based experimental validations. Therefore, one of our future aims is to apply the TIMMA model predictions to designing potential drug combination treatments, initially in various cancer cell models *in vitro*, and later also in primary samples from cancer patients *ex-vivo*. The drug treatments are also closer to the eventual translation of the model predictions in a clinical setup, at least until the RNAi-mediated target silencing has become safe and efficacious enough for clinical applications.

In an effective combinatorial setting, one needs to modulate a set of targets to achieve maximal efficacy, while avoiding others to reduce the risk of side effects. The current TIMMA algorithm addressed the first challenge: the optimal efficacy by multi-target modulation. The different model parameters and thresholds lead to a multiple candidate target inhibition networks for combinatorial treatments. From those candidate models, clinician could then ideally choose the combination that is most feasible and results in less known adverse effects, based on prior knowledge. Although there are information sources on drug side effects scattered around in databases, such as SIDER [58], ChEMBL [32], and PROMISCUOUS [59], we chose not to try to incorporate the side effect information in the current model building, because such information is still missing for many targeted drugs and the initial aim was to find effective target combinations. However, incorporating known side effect or toxicity information of drugs and their targets will be an important topic of future research. Possible approaches for such modifications include, for instance, usage of metabolic networks and pathways that are targeted by drugs [60], or combining multiple databases that contain a collection of drug features, such as medical indications, molecular targets, toxicity profiles or anatomical therapeutic and chemical classifications [61]. Further, rather than using a single response readout for drug efficacy, such as IC50, AA or DSS, the gene expression or metabolomic changes after a treatment could also be included as part of the drug response profiles, perhaps leading to be more comprehensive drug-disease networks in the future.

## Supporting Information

Dataset S1 **Binding affinity data for the CanOS1224 cell line case study.**
(XLSX)

Dataset S2 **TIMMA prediction result for the CanOS1224 data as compared to PKIM prediction.**
(XLSX)

Dataset S3 **Anticancer efficacy measured in activity area for the 15 kinase inhibitors selected from the CCLE cancer cell line drug collection.**
(XLSX)

Dataset S4 **Binding affinity data for the MCF-7, BxPC-3 and MDA-MB-231 studies.**
(XLSX)

Dataset S5 **TIMMA prediction result for MCF-7 cancer cell.**
(XLSX)

Dataset S6 **Synthetic lethality score for the targets paired with AURKB for BxPC-3 cancer cell.**
(XLSX)

Dataset S7 **TIMMA prediction result for MDA-MB-231 cancer cell.**
(XLSX)

Dataset S8 **Single and double siRNA screen data for MDA-MB-231 cancer cell.**
(XLSX)

Figure S1 **Flowchart for TIMMA model construction.**
(TIF)

Figure S2 **Flowchart of the SFFS algorithm for TIMMA model selection.**
(TIF)

Figure S3
**Concordance between TIMMA model assumptions and the actual data.** The data contains those 12 kinase inhibitors for which the quantitative binding affinity 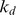 profiles across 384 kinase targets can be obtained from [41]. The drug treatment efficacy data was obtained for the CCLE collection of 504 cancer cell lines measured by Activity area [30]. The concordance index was calculated between the actual relationship between two drug efficacies (i.e. greater than or smaller than) and the model prediction using the basic subset and superset rules. The 95% confidence interval at each threshold was derived by summarizing the concordance indices for 504 cell lines. (**A**) The binary drug-target profiles were determined using a drug-specific 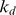 threshold defined as the n-fold of the minimal 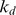 value for each drug. The feasibility of the TIMMA model assumptions is manifested by significant enhancement of the concordance index compared to random predictions (paired t-test; p-value<10^−15^). (**B**) The binary drug target profiles were determined using a fixed level of 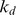 cut-off threshold. When the threshold is lower than 600 nM the model assumptions cannot be tested as none of the binarized drug-target inhibition profiles is totally inclusive of each other. At higher cut-off thresholds the model prediction performs no better than random prediction.(TIF)

Figure S4 **Synergy scores do not necessarily correlate with the single drug treatment efficacies.** (**A**) Scatter plot between synergy scores and average single drug sensitivity scores for the selected 68 drug pairs in the MDA-MB-231 study, fitted by a loess smoothing function. (**B**) Synergy scores for the selected drugs when paired with dasatinib (blue line, right axis) and the corresponding individual drug sensitivity scores (grey bars, left axis).
(TIF)

Table S1 **The top most synergistic drug pairs based on the TIMMA model predictions in MCF-7 breast cancer cells.**
(DOCX)

Table S2 **Fold-changes of the average inhibition percentages between the kinase groups in the MDA-MB-231 siRNA screen.**
(DOCX)
