# Supplementary figures and images for: Target Inhibition Networks: Predicting Selective Combinations of Druggable Targets to Block Cancer Survival Pathways
Source: PLoS Comput Biol. 2013 Sep 12;9(9):e1003226. doi: 10.1371/journal.pcbi.1003226 (PMC3772058; doi:10.1371/journal.pcbi.1003226)

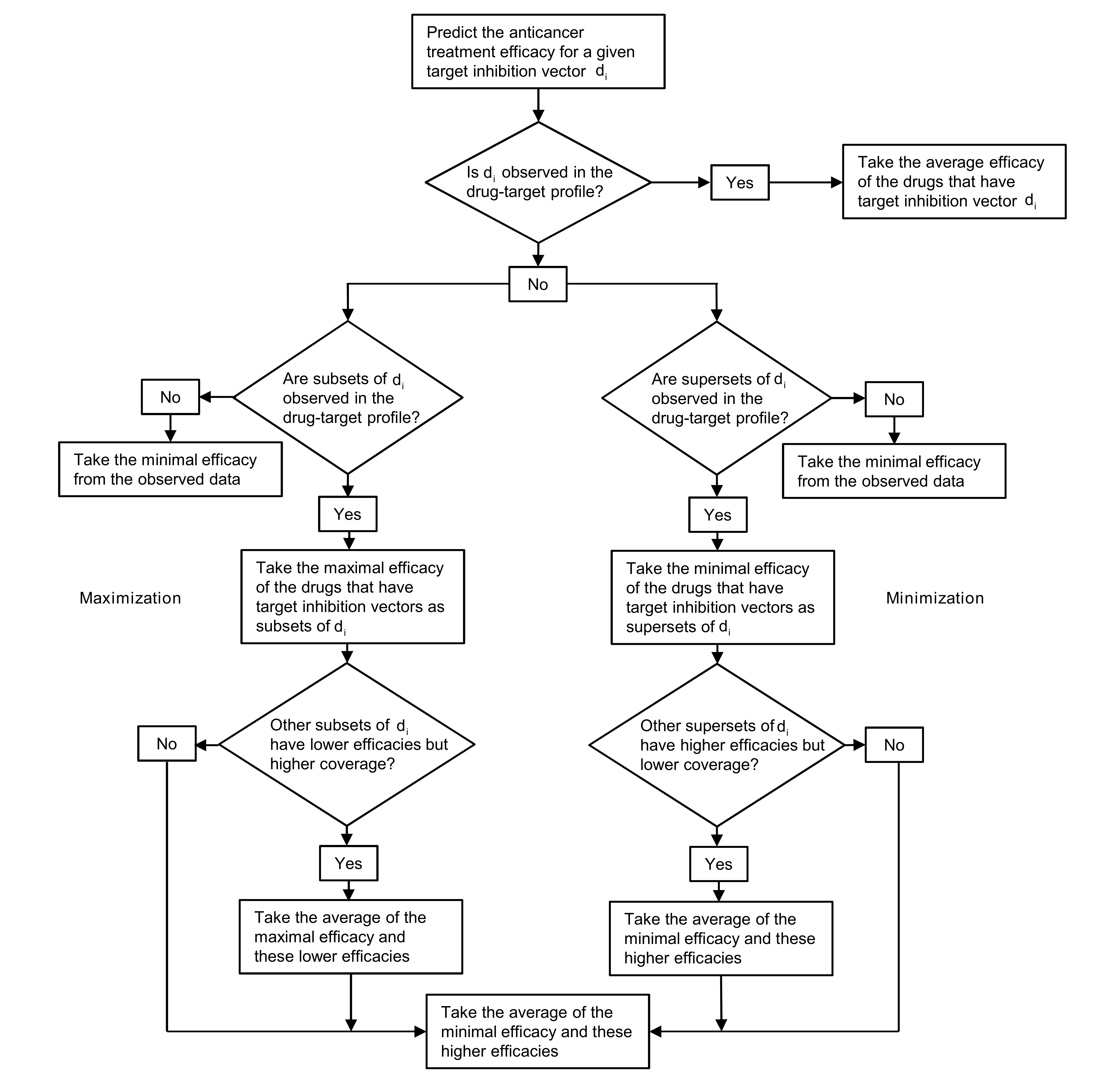

Supplement: Figure S1 — Flowchart for TIMMA model construction. (TIF) [file pcbi.1003226.s009.tif]

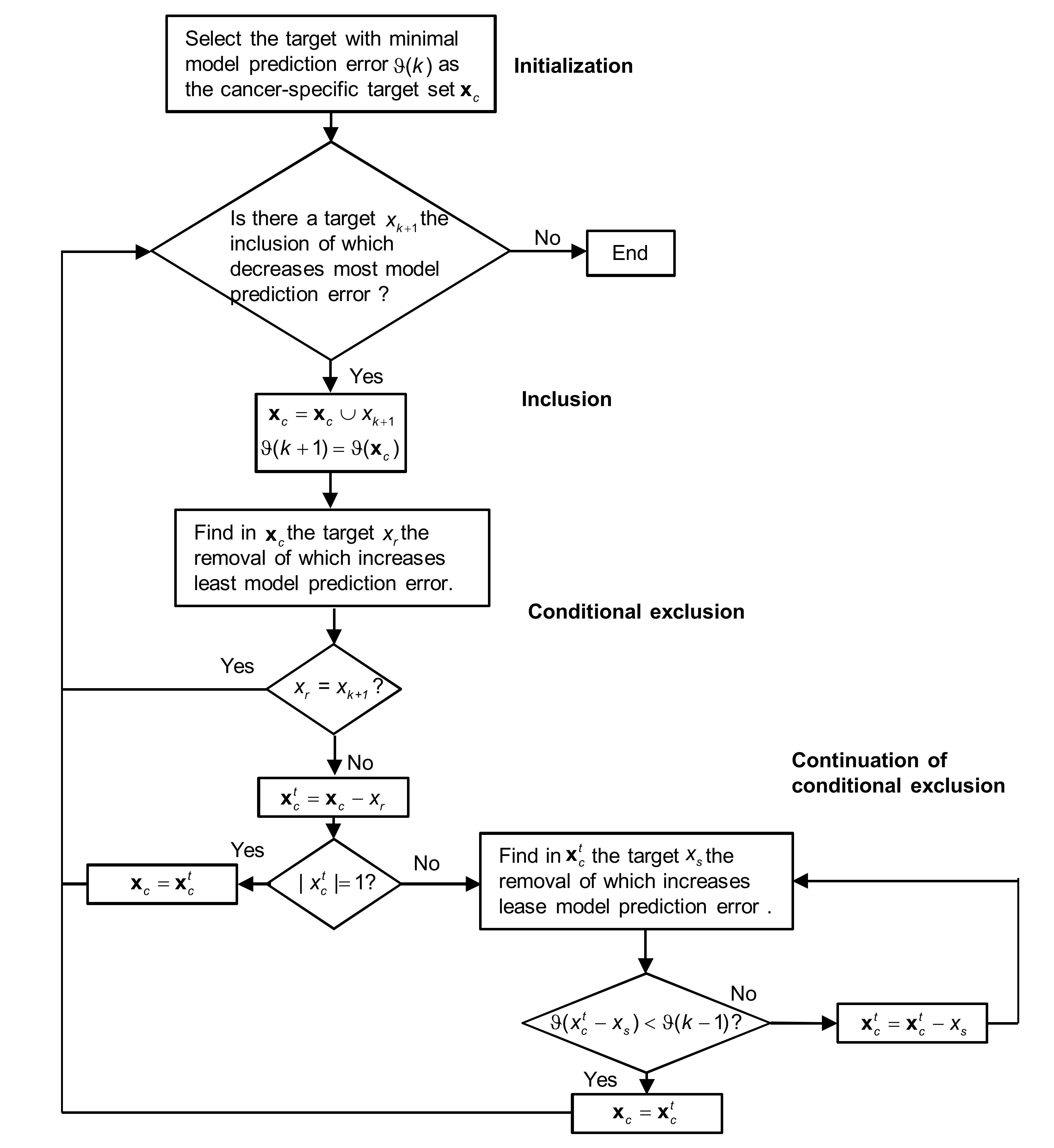

Supplement: Figure S2 — Flowchart of the SFFS algorithm for TIMMA model selection. (TIF) [file pcbi.1003226.s010.tif]

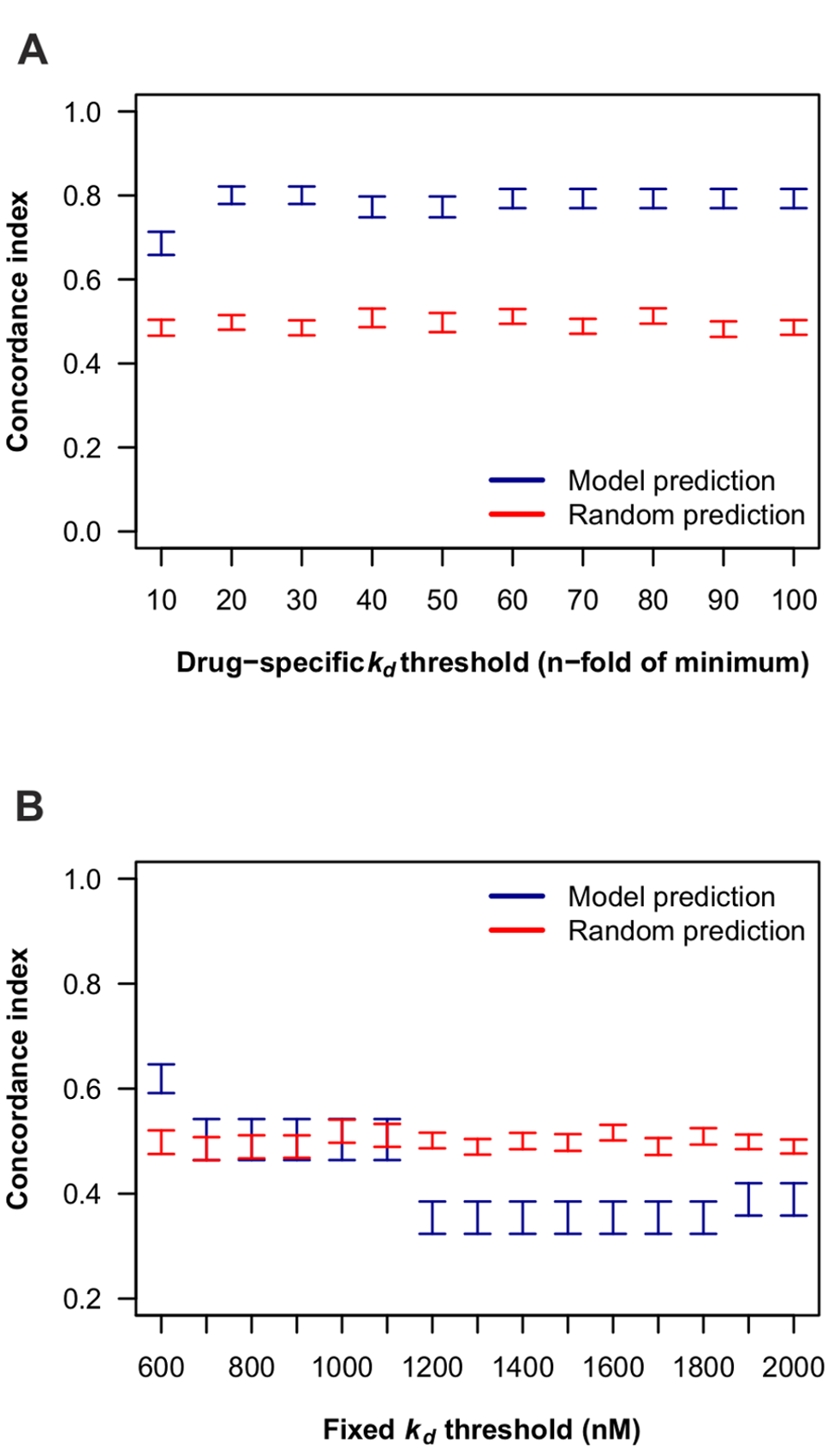

Supplement: Figure S3 — Concordance between TIMMA model assumptions and the actual data. The data contains those 12 kinase inhibitors for which the quantitative binding affinity profiles across 384 kinase targets can be obtained from [41]. The drug treatment efficacy data was obtained for the CCLE collection of 504 cancer cell lines measured by Activity area [30]. The concordance index was calculated between the actual relationship between two drug efficacies (i.e. greater than or smaller than) and the model prediction using the basic subset and superset rules. The 95% confidence interval at each threshold was derived by summarizing the concordance indices for 504 cell lines. (A) The binary drug-target profiles were determined using a drug-specific threshold defined as the n-fold of the minimal value for each drug. The feasibility of the TIMMA model assumptions is manifested by significant enhancement of the concordance index compared to random predictions (paired t-test; p-value<10−15). (B) The binary drug target profiles were determined using a fixed level of cut-off threshold. When the threshold is lower than 600 nM the model assumptions cannot be tested as none of the binarized drug-target inhibition profiles is totally inclusive of each other. At higher cut-off thresholds the model prediction performs no better than random prediction. (TIF) [file pcbi.1003226.s011.tif]

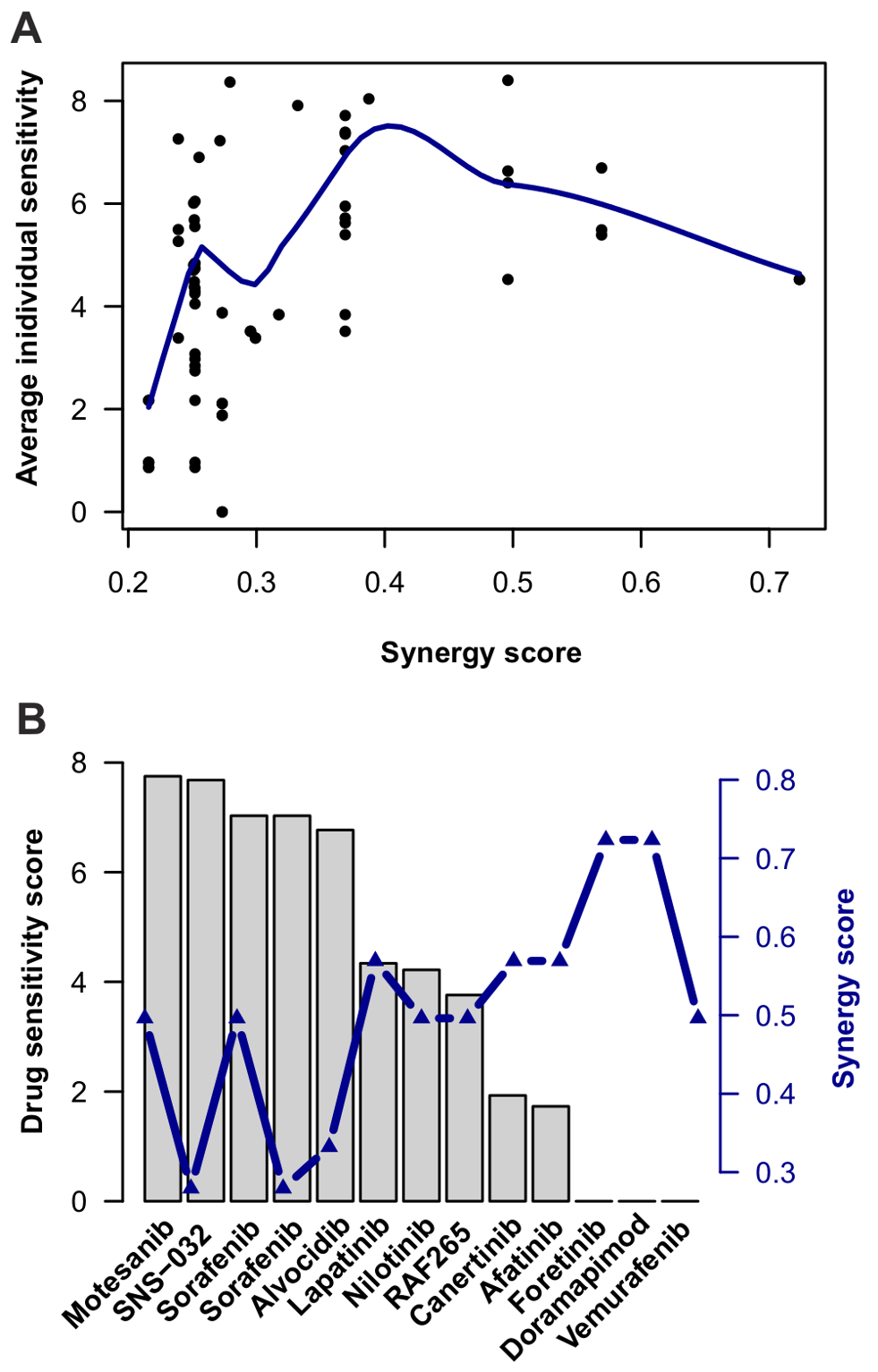

Supplement: Figure S4 — Synergy scores do not necessarily correlate with the single drug treatment efficacies. (A) Scatter plot between synergy scores and average single drug sensitivity scores for the selected 68 drug pairs in the MDA-MB-231 study, fitted by a loess smoothing function. (B) Synergy scores for the selected drugs when paired with dasatinib (blue line, right axis) and the corresponding individual drug sensitivity scores (grey bars, left axis). (TIF) [file pcbi.1003226.s012.tif]
