# Supplementary material for: Target Inhibition Networks: Predicting Selective Combinations of Druggable Targets to Block Cancer Survival Pathways
Source: PLoS Comput Biol. 2013 Sep 12;9(9):e1003226. doi: 10.1371/journal.pcbi.1003226 (PMC3772058; doi:10.1371/journal.pcbi.1003226)
Supplement: Table S1 — The top most synergistic drug pairs based on the TIMMA model predictions in MCF-7 breast cancer cells. (DOCX) [file pcbi.1003226.s013.docx]

**Table S1. The top most synergistic drug pairs based on the TIMMA model predictions in MCF-7 breast cancer cells.**

| **Drug pair** | **AA_1_** | **AA_2_** | **Predicted efficacy** | **Synergy score** |
| --- | --- | --- | --- | --- |
| Crizotinib Erlotinib | 0.357 | 0.502 | 1.000 | 0.928 |
| Crizotinib TAE-684 | 1.578 | 0.502 | 1.000 | 0.928 |
| Erlotinib TAE-684 | 0.357 | 1.578 | 1.000 | 0.928 |
| Crizotinib Vandetanib | 0.216 | 0.502 | 0.545 | 0.478 |
| TAE-684 Vandetanib | 0.761 | 1.033 | 0.404 | 0.345 |
| Dovitinib Vemurafenib | 0.581 | 0.372 | 0.368 | 0.336 |
| Selumetinib CHIR-265/RAF-265 | 0.015 | 0.582 | 0.369 | 0.328 |
| Selumetinib Sorafenib | 0.000 | 0.582 | 0.369 | 0.328 |
| CHIR-265/RAF-265 PD-0325901 | 0.015 | 0.362 | 0.369 | 0.328 |
| PD-0325901 Sorafenib | 0.000 | 0.362 | 0.369 | 0.328 |
| Selumetinib Nilotinib | 0.124 | 0.582 | 0.369 | 0.324 |
| Nilotinib PD-0325901 | 0.124 | 0.362 | 0.369 | 0.324 |
| Selumetinib Vemurafenib | 0.581 | 0.582 | 0.369 | 0.313 |
| PD-0325901 Vemurafenib | 0.581 | 0.362 | 0.369 | 0.313 |
| Selumetinib Vandetanib | 0.216 | 0.582 | 0.369 | 0.305 |
| PD-0325901 Vandetanib | 0.216 | 0.362 | 0.369 | 0.305 |
| Selumetinib Erlotinib | 0.357 | 0.582 | 0.369 | 0.301 |
| Erlotinib PD-0325901 | 0.357 | 0.362 | 0.369 | 0.301 |
| Selumetinib Dovitinib | 0.372 | 0.582 | 0.369 | 0.298 |
| Dovitinib PD-0325901 | 0.372 | 0.362 | 0.369 | 0.298 |
| Selumetinib TAE-684 | 1.578 | 0.582 | 0.369 | 0.287 |
| PD-0325901 TAE-684 | 1.578 | 0.362 | 0.369 | 0.287 |
| Dovitinib CHIR-265/RAF-265 | 0.015 | 0.372 | 0.310 | 0.278 |
| Dovitinib Sorafenib | 0.000 | 0.372 | 0.310 | 0.278 |
| CHIR-265/RAF-265 Crizotinib | 0.015 | 0.502 | 0.318 | 0.275 |
| Crizotinib Sorafenib | 0.000 | 0.502 | 0.318 | 0.275 |
| Selumetinib Crizotinib | 0.502 | 0.582 | 0.369 | 0.273 |
| Crizotinib PD-0325901 | 0.502 | 0.362 | 0.369 | 0.273 |

AA_1/2_, measured treatment efficacy (activity area) when using either of the drugs alone; Predicted efficacy, predicted treatment efficacy by the TIMMA model. Synergy score, drug synergy score calculated by (Eq. 10). The table lists the drug pairs with synergy scores higher than the median (0.272) of the total population (n=60).
