# Supplementary material for: Target Inhibition Networks: Predicting Selective Combinations of Druggable Targets to Block Cancer Survival Pathways
Source: PLoS Comput Biol. 2013 Sep 12;9(9):e1003226. doi: 10.1371/journal.pcbi.1003226 (PMC3772058; doi:10.1371/journal.pcbi.1003226)
Supplement: Table S2 — Fold-changes of the average inhibition percentages between the kinase groups in the MDA-MB-231 siRNA screen. (DOCX) [file pcbi.1003226.s014.docx]

**Table S2. Fold-changes of the average inhibition percentages between the kinase groups in the MDA-MB-231 siRNA screen.**

|  | **Kinome-wide** | **Single** | **High** |
| --- | --- | --- | --- |
| **Single** | 1.22 (1.000) |  |  |
| **High** | 1.80 (<10^-15^) | 1.48 (0.013) |  |
| **Low** | 1.47 (3.4e-07) | 1.28 (0.707) | 1.23 (0.031) |

Kinome-wide, single kinases in the kinome-wide background; Single, single targets selected by TIMMA; High/Low, target pairs in the TIMMA selection with predicted efficacies above/below 0.6. P-values after Bonferroni adjustment are reported in the parentheses.
